# Predicting p*K*_a_ Values of Para-Substituted Aniline Radical Cations vs. Stable Anilinium Ions in Aqueous Media

**DOI:** 10.3390/molecules29194522

**Published:** 2024-09-24

**Authors:** Jingxin Wang, Hansun Fang, Zixi Zhong, Huajun Huang, Ximei Liang, Yufan Yuan, Wenwen Zhou, Davide Vione

**Affiliations:** 1 Guangdong Provincial Engineering Technology Research Center of Public Health Detection and Assessment, School of Public Health, Guangdong Pharmaceutical University, Guangzhou 510310, China; wjxdaxue@163.com; 2 Key Laboratory of Poyang Lake Basin Agricultural Resource and Ecology of Jiangxi Province, College of Land Resource and Environment, Jiangxi Agricultural University, Nanchang 330045, China; huanghuajun2004@126.com (H.H.); 13767258573@163.com (Y.Y.); wenwenzhou@jxau.edu.cn (W.Z.); 3 AP&A-Level Program, Guangzhou Foreign Language School, Guangzhou 511455, China; zhongzixi2007@163.com; 4 College of Animal Science and Technology, Jiangxi Agricultural University, Nanchang 330045, China; willie3@163.com; 5 Dipartimento di Chimica, Università di Torino, Via P. Giuria 5, 10125 Torino, Italy

**Keywords:** aniline, radical cation, cation, p*K*_a_, quantum chemical calculation

## Abstract

The focus of p*K*_a_ calculations has primarily been on stable molecules, with limited studies comparing radical cations and stable cations. In this study, we comprehensively investigate models with implicit solvent and explicit water molecules, direct and indirect calculation approaches, as well as methods for calculating free energy, solvation energy, and quasi-harmonic oscillator approximation for para-substituted aniline radical cations (R-PhNH_2_^•+^) and anilinium cations (R-PhNH_3_^+^) in the aqueous phase. Properly including and positioning explicit H_2_O molecules in the models is important for reliable p*K*_a_ predictions. For R-PhNH_2_^•+^, precise p*K*_a_ values were obtained using models with one or two explicit H_2_O molecules, resulting in a root mean square error (RMSE) of 0.563 and 0.384, respectively, for both the CBS-QB3 and M062X(D3)/ma-def2QZVP methods. Further improvement was achieved by adding H_2_O near oxygen-containing substituents, leading to the lowest RMSE of 0.310. Predicting p*K*_a_ values for R-PhNH_3_^+^ was more challenging. CBS-QB3 provided an RMSE of 0.349 and the M062X(D3)/ma-def2QZVP method failed to calculate p*K*_a_ accurately (RMSE > 1). However, by adopting the double-hybrid functional method and adding H_2_O near the R substituent group, the calculations were significantly improved with an average absolute difference (Δp*K*_a_) of 0.357 between the calculated and experimental p*K*_a_ values. Our study offers efficient and reliable methods for p*K*_a_ calculations of R-PhNH_2_^•+^ (especially) and R-PhNH_3_^+^ based on currently mature quantum chemistry software.

## 1. Introduction

The determination of acid–base dissociation equilibrium constants (p*K*_a_ values) is essential for studying the physicochemical properties and environmental fate of compounds in the aqueous phase, as changes in their protonation forms can significantly impact reaction rates, pathways, and mechanisms [1]. Traditionally, the p*K*_a_ value is determined by electrochemical methods [2] or by analyzing the absorption spectra of a compound under different pH conditions [3]. However, conducting p*K*_a_ measurements can be influenced by various factors. For instance, handling toxic compounds can be challenging, and impurities in the target compound may interfere with the measurement of absorption spectra [4]. Moreover, compounds with multiple ionization groups may exhibit multiple p*K*_a_ values that are difficult to distinguish solely through absorption spectroscopy [5,6].

When measuring the p*K*_a_ of compounds with short lifetimes, such as organic and inorganic radicals, compared to stable chemicals, additional challenges arise. Normally, determining the p*K*_a_ values of short-lived species relies on measuring changes in transient absorption spectra, typically using methods like laser flash photolysis or pulse radiolysis [7,8]. However, these techniques can be costly, and their sensitivity depends on both the detector and the emission spectral intensity of the probe light, especially in the ultraviolet range. Insufficient probe photons may lead to neglect of important peaks in the short-wavelength range [9], which could be critical for accurate p*K*_a_ determinations.

In recent years, research has shown that p*K*_a_ values can be precisely determined by quantum chemical (QC) calculations [10,11]. The critical factor for achieving accurate calculation results primarily lies in choosing an appropriate solvent model [12,13]. Currently, the density-based solvation model (SMD) is widely employed during p*K*_a_ calculations. SMD is a continuum solvation model that relies on the quantum mechanical charge density of a solute molecule interacting with a continuum description of the solvent [14].

Several general methods in conjunction with SMD have been employed to increase the accuracy of p*K*_a_ calculations in the aqueous phase. Firstly, the utilization of direct and indirect approaches. The direct approach involves calculating p*K*_a_ values directly with solvent effects already incorporated in the molecular models, while the indirect approach relies on thermodynamic cycles between the gas and solvent phases [15,16]. Another method is the inclusion of explicit solvent molecules in the molecular models [17,18,19], although this may increase calculation time as the number of solvent molecules grows. Moreover, careful selection and utilization of specific calculation methods and basis sets for different forms of targeted compounds (e.g., neutral, cationic, and anionic molecules) can be helpful [20,21]. In addition, professional researchers have also developed advanced strategies, such as modifying the solute cavity [22,23,24] and creating new solvent models that better describe weak interaction forces between molecules [25]. However, the practical application of these advanced methods requires a profound understanding of (and experience with) the underlying theory. For example, cavity adjustments were found to be sensitive to molecular modes and the type of solvents used, and it is still uncertain whether the same rules can be applied across different systems [20]. Therefore, from the perspective of experimental chemists, and especially those focused on specific classes of compounds, a more practical and attractive approach to predict p*K*_a_ values involves utilizing pre-existing QC and data correction software, as well as the built-in solvent models, methods, and basis sets.

Aniline (-PhNH_2_) is a chemical group present in various agricultural, medical, and industrial compounds and natural dissolved organic matter (NDOM) [26,27,28,29,30]. The protonation of -PhNH_2_ significantly influences the charge and state of the compounds. Additionally, the electron-rich nature of -PhNH_2_ enables it to act as an electron donor and initiate redox reactions, resulting in the formation of a radical cation (R-PhNH_2_^•+^, where R represents the substituent group) [31,32]. The R-PhNH_2_^•+^ species can either accept electrons from other reductive compounds or undergo deprotonation to form neutral radicals (R-PhNH^•^). R-PhNH_2_^•+^ tends to act as a potent electron acceptor, which can be reduced again to regenerate R-PhNH_2_ [33], while R-PhNH^•^ might undergo intramolecular reactions through rearrangement [31].

Pulse radiolysis studies have revealed that the average p*K*_a_ value of para-substituted R-PhNH_2_^•+^ is 7.3 [7], which is within the typical pH range found in natural water bodies. In contrast, the corresponding anilinium cations (R-PhNH_3_^+^) have an average p*K*_a_ value of only 3.9 (Table 1) [34]. These findings suggest that, in the natural aqueous environment, the transformation pathways and kinetics of R-PhNH_2_^•+^ would be more sensitively influenced by the p*K*_a_ value in comparison with R-PhNH_3_^+^.

There have been studies reporting and calculating the p*K*_a_ values of R-PhNH_2_^•+^ in organic solvents, such as DMSO [49,50]. However, there is limited information available on the calculation and prediction of p*K*_a_ values of R-PhNH_2_^•+^ in water. Therefore, the main objective of this study is to identify a suitable QC method for accurately determining p*K*_a_ based on the SMD model. Additionally, p*K*_a_ values for R-PhNH_3_^+^ were also evaluated for comparison. Through a systematic study of p*K*_a_ calculation methods for both R-PhNH_2_^•+^ and R-PhNH_3_^+^, this research provides a practical tool for comprehensive prediction of the transformation of R-PhNH_2_ in aqueous environments, and it also offers valuable insights into the ideas and approaches relevant to calculating p*K*_a_ values of similar compounds.

## 2. Results and Discussion

### 2.1. Calculation of pKa

All quantum chemical calculations were performed with Gaussian 16 [51]. M062X(D3)/6-311++g(d,p), coupled with the IEFPCM solvent model, was selected as the optimization method [52,53,54,55,56]. For radicals, the Unrestricted Hartree–Fock (UHF) formalism was used, while the Restricted Hartree–Fock (RHF) formalism was applied for anilines. For compounds containing heavy atoms, such as iodine (I) in this study, a mixed strategy was adopted for molecular optimization. Specifically, light atoms belonging to the first four periods of elements were optimized using the 6-311++g(d,p) basis set, while the iodine (I) atom was optimized using the ma-def2TZVP basis set [57,58]. The geometries obtained by these methods were then used for all subsequent calculations. For the deprotonation of R-PhNH_2_^•+^ and R-PhNH_3_^+^, p*K*_a_ values were calculated according to Equation (1) [17].

(1) $$pK_{a}=\frac{\Delta G_{\mathrm{deprot}}(\mathrm{sol})}{2.303RT}$$

where Δ*G*_deprot_(sol) represents the free energy change for the deprotonation of compounds in the aqueous phase. Δ*G*_deprot_(sol) was calculated by both direct and indirect (thermodynamic) approaches, as described in Section 3.1. *R* denotes the ideal gas constant (8.314 J mol^−1^ K^−1^), and *T* represents the ambient temperature set at 298.15 K.

The complete basis set method CBS-QB3 is considered as an accurate and reliable method for calculating *G*(sol) [16,59]. However, it is not applicable to compounds containing heavy atoms such as I-PhNH_2_^•+^ and I-PhNH_3_^+^, as investigated herein. To address this limitation, Δ*G*_deprot_(sol) was also calculated based on the method and basis set of M062X(D3)/ma-def2QZVP [52,57,58].

### 2.2. Numbers and Positions of Explicit Water Molecules in the Models

The effects of the addition of one to four explicit water (H_2_O) molecules were examined in the deprotonation of R-PhNH_2_^•+^ (one to four H_2_O molecules) and R-PhNH_3_^+^ (one to three H_2_O molecules). When constructing the molecular models, H_2_O molecules were positioned around the amino group (-NH_x_). The maximum number of added H_2_O molecules was determined based on the hydrogen bonds that could possibly be formed between -NH_x_ and the H_2_O molecules. Some of the H_2_O molecules connected with -NH_x_ were presumed to be hydrogen donors, while others served as electron-pair donors (Figure S1).

**Figure 1:**
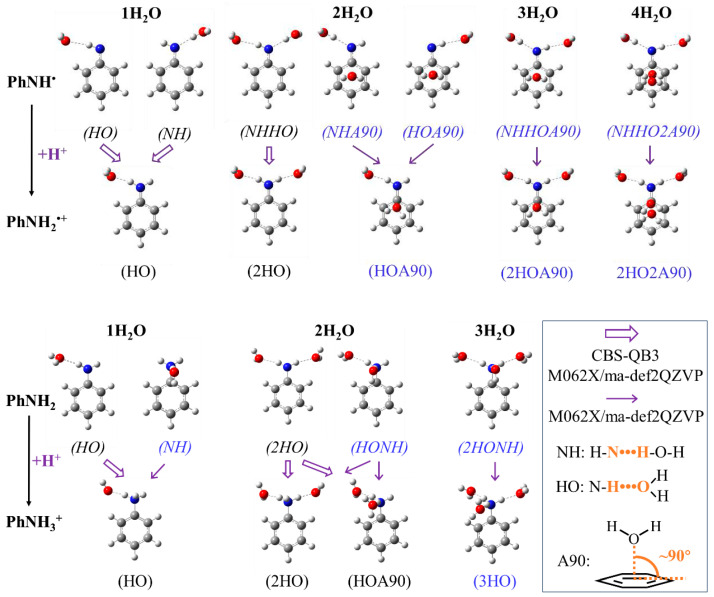
The optimized structures of H-PhNH^•^, H-PhNH_2_^•+^, H-PhNH_2_, and H-PhNH_3_^+^ with 1–4 explicit H_2_O molecules at different positions. Labels HO and NH indicate that the amino group forms a hydrogen bond with H_2_O as a H donor and electron-pair (nitrogen) donor, respectively. A90 means one H_2_O molecule is positioned approximately perpendicular to the plane of the benzene ring of PhNH_2_. Italic font indicates the molecules in deprotonated form. Blue color indicates that the calculations of free energy were solely based on M062X(D3)/ma-def2QZVP and not on CBS-QB3.

Figure 1 presents a group of optimized models illustrating the (de)protonated states of H-PhNH^•^/H-PhNH_2_^•+^ and H-PhNH_2_/H-PhNH_3_^+^ as a typical example. It is notable that some models undergo significant changes or fail to meet convergence criteria when calculated based on the CBS-QB3 method (labeled in blue in Figure 1). This is primarily due to the readjustments made to the positions of H_2_O molecules during optimization with the CBS-QB3 method. Consequently, the final molecular models were significantly different from the originally optimized structures based on M062X(D3)/6-311++g(d,p).

Examples illustrating the structural changes when employing the CBS-QB3 method can be found in Figures S2 and S3. Therefore, for these models, *G*(sol) and *G*(gas) were solely calculated based on M062X(D3)/ma-def2QZVP.

### 2.3. Impact of the Number of Explicit H_2_O Molecules on pK_a_ Calculations

**Figure 2:**
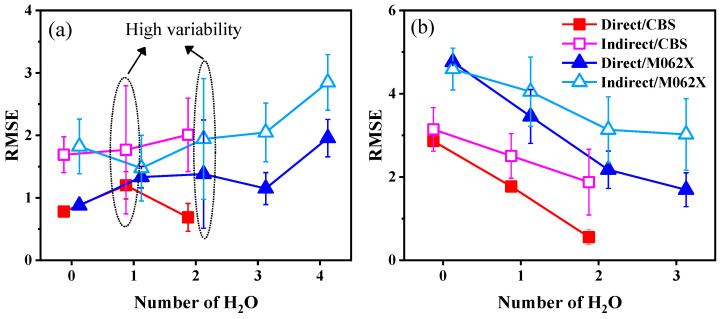
Impact of the numbers of H_2_O molecules on p*K*_a_ calculations for (**a**) R-PhNH_2_^•+^ and (**b**) R-PhNH_3_^+^ based on CBS-QB3 and M062X/ma-def2QZVP methods with direct and indirect approaches.

All calculated energies, as well as the experimental results and calculated p*K*_a_ values of R-PhNH_2_^•+^ and R-PhNH_3_^+^ in the aqueous phase, are summarized and compared in Tables S1–S6 in the Supporting Materials. The impact of the number of explicit H_2_O molecules on p*K*_a_ calculations was assessed based on the variation of root mean square error (RMSE) as shown in Figure 2. For both R-PhNH_2_^•+^ and R-PhNH_3_^+^, the CBS-QB3 method was found to be appropriate for models containing up to two H_2_O molecules.

However, if the number of H_2_O molecules was three or higher, using the CBS-QB3 method for further energy calculations would lead to significant structural changes from the originally optimized models (Figures S2 and S3).

The lowest average values of root mean square error (RMSE_aver_) for R-PhNH_2_^•+^ and R-PhNH_3_^+^ were 0.687 and 0.557, respectively, demonstrating that the p*K*_a_ values calculations of R-PhNH_2_^•+^ were comparable in performance to those of R-PhNH_3_^+^. Moreover, when comparing models with and without H_2_O molecules, the largest differences in RMSE_aver_ for R-PhNH_2_^•+^ and R-PhNH_3_^+^ were 1.211 and 3.067, respectively, indicating that adding H_2_O molecules is an efficient strategy to improve the results of p*K*_a_ calculations [17,60], especially for R-PhNH_3_^+^. The RMSE_aver_ value for R-PhNH_3_^+^ consistently decreased with an increase in the number of H_2_O molecules in the models, indicating a corresponding increase in calculation accuracy. Always in the case of R-PhNH_3_^+^, the CBS-QB3 method exhibited notably improved performance upon addition of H_2_O molecules compared to the M062X(D3)/ma-def2QZVP method.

In the case of R-PhNH_2_^•+^, the variation in calculation accuracy showed a more complex pattern. With the direct approach, the calculation accuracy initially decreased (i.e., RMSE_aver_ increased) when adding only one H_2_O molecule. As more H_2_O molecules were included in the models, the calculation accuracy subsequently improved until the number of H_2_O molecules exceeded three, in which case the calculation accuracy decreased (RMSE_aver_ increased) once again. Conversely, different results were obtained with the indirect approach. With the exception of adding a single H_2_O molecule based on the M062X(D3)/ma-def2QZVP method, the inclusion of additional H_2_O molecules generally decreased calculation accuracy with the indirect approach, as evidenced by an increase in RMSE_aver_.

When assessing the accuracy of calculation methods, it is crucial not to rely solely on RMSE_aver_. Instead, it is equally important to consider the standard deviation of RMSE (RMSE_std_). Notably, in models of R-PhNH_2_^•+^ (Figure 2a), there was a significant increase in RMSE_std_ in the case of one (RMSE_std_ = ±1.025) and two H_2_O molecules (RMSE_std_ = ±0.967). The large span of RMSE_std_ values obtained from these models of R-PhNH_2_^•+^ suggests that there is room for further improving p*K*_a_ calculations, for instance, by further modifying the models and adjusting the calculation parameters. In contrast, much lower RMSE_std_ values were obtained with R-PhNH_3_^+^ (ranging from ±0.017 to ±0.861), possibly indicating a more limited space for improvement of calculations based on the present approaches and methods.

### 2.4. Impact of H_2_O Molecule Positions on pK_a_ Calculation

**Figure 3:**
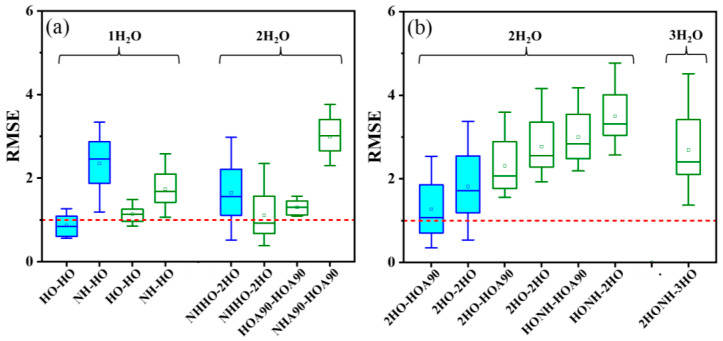
Impact of the H_2_O positions (x-axis and the meanings of the labels are referred to Figure 1) in models on p*K*_a_ calculations of (**a**) R-PhNH_2_^•+^ and (**b**) R-PhNH_3_^+^ based on CBS-QB3 (blue and filled boxes) and M062X(D3)/ma-def2QZVP methods (green and unfilled boxes). The red dashed lines indicate RMSE = 1.

The positions of explicit H_2_O molecules have rarely been considered in previous studies. Here, in addition to the number of H_2_O molecules, the influence of their relative positions to the amino group on the accuracy of p*K*_a_ calculations was also studied, based on models with low RMSE_aver_ (indicating high calculation accuracy) or with high RMSE_std_ (indicating potential for improvement), as obtained in the last section. Specifically, we considered R-PhNH_2_^•+^ models with one and two H_2_O molecules, as well as R-PhNH_3_^+^ models with two and three H_2_O molecules (Figure 3).

Regarding R-PhNH_2_^•+^ (Figure 3a), introducing a H_2_O molecule placed approximately perpendicular to the plane of the benzene ring would result in decreased accuracy. Based on the CBS-QB3 method, the best calculation results were achieved with models containing a single H_2_O molecule, which acts as an electron-couple donor for both R-PhNH^•^ and R-PhNH_2_^•+^ (both in the form of HO structure as HO-HO, see Figure 1). In this model, the values of RMSE_aver_ ± RMSE_std_ were 0.865 ± 0.260.

As for the M062X(D3)/ma-def2QZVP method, the best results were obtained with models containing two H_2_O molecules. H_2_O and R-PhNH^•^ were constructed according to the NHHO structure, while H_2_O and R-PhNH_2_^•+^ were built based on the 2HO structure (NHHO-2HO, see Figure 1 for these structures). In this case, we obtained RMSE_aver_ ± RMSE_std_ = 1.113 ± 0.616. The high ratios of RMSE_std_ to RMSE_aver_ (~55%) suggest that the present models with optimized numbers and positions of H_2_O molecules could still be significantly modified to increase calculation accuracy.

Regarding R-PhNH_3_^+^ (Figure 3b), the lowest RMSE was obtained with the CBS-QB3 method with two H_2_O molecules. H_2_O and R-PhNH_2_ were constructed according to the 2HO structure, while H_2_O and R-PhNH_3_^+^ were built based on the HOA90 structure (2HO-HOA90, see Figure 1 for these structures). In the case of the M062X(D3)/ma-def2QZVP method, the best model includes three H_2_O molecules positioned in the 2HONH structure for R-PhNH_2_ and in the 3HO structure for R-PhNH_3_^+^ (2HONH-3HO). However, the RMSE values obtained from all models based on the M062X(D3)/ma-def2QZVP method exceeded 1.

### 2.5. Combination of Optimal Models and Methods for pK_a_ Calculation

**Figure 4:**
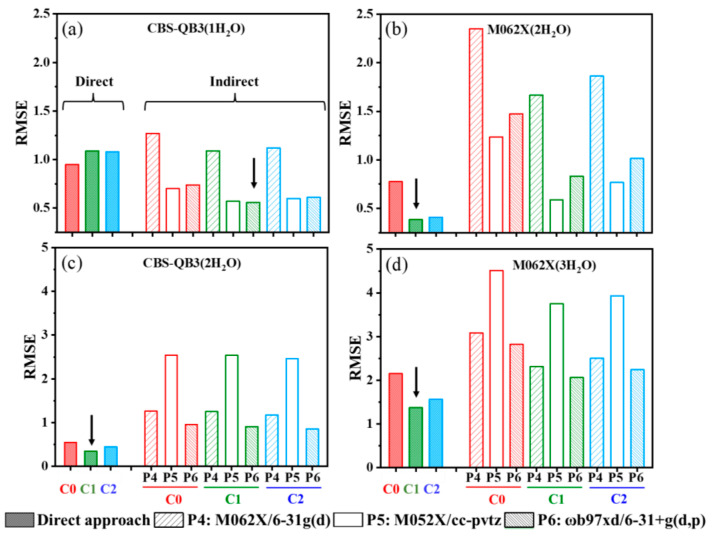
The values of RMSE were calculated for different combinations of calculation approaches (direct and indirect), correction methods (C0: rigid-rotor harmonic oscillator method; C1,C2: two different quasi-rigid-rotor harmonic oscillator methods; see Section 3.1), and protocols for calculating solvation energy (P4–P6, see calculation methods in Section 3.1.2). Figures (**a**,**b**) present the results for R-PhNH_2_^•+^, while figures (**c**,**d**) depict the results for R-PhNH_3_^+^. The arrows indicate the combination of specific approaches and methods that achieved the highest accuracy in each case (lowest RMSE).

To improve the p*K*_a_ calculation accuracy based on the current methods and basis sets, three methodological aspects were considered: (i) choosing appropriate approaches; (ii) employing suitable correction methods; and (iii) selecting proper protocols for calculating solvation energies (only for the indirect approach). To determine the best combinations of optimal models and methods, the RMSE values obtained from different combinations, based on the established optimized models, are summarized and compared in Figure 4.

Overall, for both R-PhNH_2_^•+^ and R-PhNH_3_^+^, the CBS-QB3 method emerged as the most accurate choice, which has been widely suggested for p*K*_a_ calculations in several reports [16,61]. For R-PhNH_2_^•+^, the quasi-rigid-rotor harmonic oscillator (QRRHO) correction method (C1 and C2, see Section 3.1) effectively improved calculation results, with C1 slightly outperforming C2 in the case of the M062X method. For calculations through the indirect approach, the choice of an appropriate protocol for calculating solvation energy played a more essential role in improving accuracy than the correction method. For R-PhNH_2_^•+^, M062X/6-31G(d) (P4) produced the least accurate results, whereas for R-PhNH_3_^+^, M052X/cc-pvtz (P5) was the least effective. In most cases, ωb97xd/6-31+g(d,p) (P6) delivered the best performance.

Once the correction method (C1) and protocol for calculating solvation energy (ωb97xd/6-31+g(d,p) in most cases, i.e., P6), were determined, the calculation errors were significantly minimized, regardless of whether the direct or indirect approach was adopted. Specifically, for R-PhNH_2_^•+^, the best combination was the indirect approach/CBS-QB3/C1/P6 based on models with one H_2_O molecule (both R-PhNH^•^ and R-PhNH_2_^•+^ in the HO structure), resulting in an RMSE of 0.563. For calculating p*K*_a_ of R-PhNH_2_^•+^ containing heavy atoms, the best combination was the direct approach/M062X(D3)/ma-def2QZVP/C1 based on models with two H_2_O molecules (R-PhNH^•^ in the NHHO structure and R-PhNH_2_^•+^ in the 2HO structure), which gave an RMSE of 0.384.

These results suggest that precise aqueous p*K*_a_ predictions would be achieved for R-PhNH_2_^•+^ based on either the CBS-QB3 or the M062X(D3)/ma-def2QZVP method. From a practical standpoint, the direct approach combined with the M062X(D3)/ma-def2QZVP method holds advantages as it predicts the p*K*_a_ of molecules containing heavy atoms without additional steps for calculating solvation energy. However, it is imperative to note that the indirect approach utilizing CBS-QB3/C1/P6 keeps the calculation time within merely 94 min (H-PhNH_2_^•+^ as a typical example), while the direct approach with M062X(D3)/ma-def2QZVP/C1 requires a notably longer calculation time of 626 min (Figure S4a).

For R-PhNH_3_^+^, the optimal combination of methods was the direct approach/CBS-QB3/C1 based on the model with two H_2_O molecules (H_2_O and R-PhNH_2_ were constructed according to the 2HO structure, while H_2_O and R-PhNH_3_^+^ were built based on the HOA90 structure), resulting in an RMSE of 0.349 that is comparable to the RMSE of R-PhNH_2_^•+^ (0.563). Moreover, the RMSE obtained with the M062X(D3)/ma-def2QZVP method was 1.375 (direct approach/C1, models with three H_2_O molecules), indicating an inability to accurately predict the p*K*_a_ values of molecules containing heavy atoms. These results suggest the p*K*_a_ calculation is more difficult for R-PhNH_3_^+^ than for R-PhNH_2_^•+^. New strategies will thus be explored to predict the p*K*_a_ of R-PhNH_3_^+^, rather than just selecting and modifying the current calculation methods that proved satisfactory for R-PhNH_2_^•+^. Moreover, the calculation time for the direct approach employing CBS-QB3/C1 is relatively short, taking only 69 min (H-PhNH_3_^+^ as a typical example), whereas the direct approach with M062X(D3)/ma-def2QZVP/C1 requires a significantly longer duration of 501 min (Figure S4b).

### 2.6. Strategies for Further Improvements of pK_a_ Calculations

**Figure 5:**
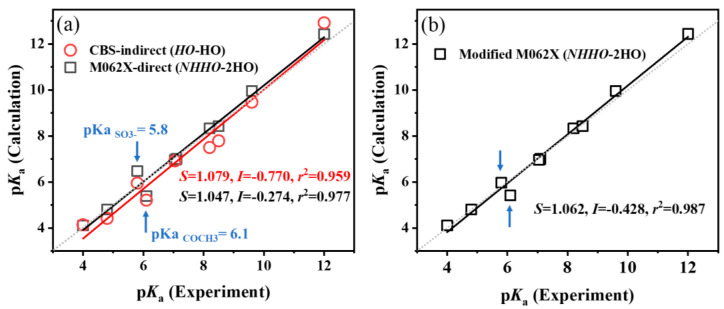
Validation of the performance of different combinations of models and methods on p*K*_a_ calculations of R-PhNH_2_^•+^. Calculated p*K*_a_ values (**a**) before and (**b**) after modifications. The dotted lines indicate the ideal results with *S* = 1 and *I* = 0. Arrows indicate the models modified by adding extra H_2_O molecules.

**Figure 6:**
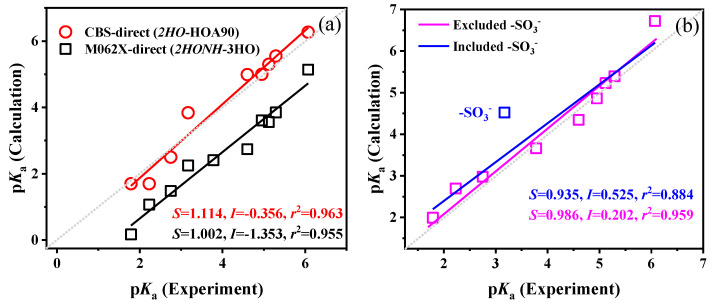
Validation of the performance of different combinations of models and methods on p*K*_a_ calculations of R-PhNH_3_^+^. Calculated p*K*_a_ values were based on (**a**) CBS-QB3 with two H_2_O molecules in models and M062X(D3)/ma-def2QZVP with three H_2_O molecules in models; (**b**) revDSD-PBEP86-D3(BJ)/ma-def2QZVPP method with three H_2_O molecules around amino and extra H_2_O near R group for formation of hydrogen bonds (correction method: C0). The dotted lines indicate the ideal results with *S* = 1 and *I* = 0.

Adopting a mixed calculation strategy can potentially lead to significant improvements in the accuracy of p*K*_a_ calculations. Instead of employing the same strategy for all compounds, adopting different strategies for specific molecular structures might yield better overall results [20]. To explore this possibility and identify potentially specific structures among the ten compounds we investigated, a comparison was made between the experimental and calculated p*K*_a_ values in the aqueous phase, the latter obtained from the best combination of models and methods for R-PhNH_2_^•+^ and R-PhNH_3_^+^ (Figure 5 and Figure 6).

For R-PhNH_2_^•+^, both the CBS-QB3 and M062X(D3)/ma-def2QZVP methods produced regression lines with slopes (*S*) close to 1, intercepts (*I*) close to 0, and correlation coefficients (*r*^2^) > 0.959 (Figure 5a). Therefore, both regression lines were precise for calculating the p*K*_a_ values of R-PhNH_2_^•+^. However, upon closer examination, it was revealed that the largest errors in the linear regressions originated from the p*K*_a_ values associated with compounds containing negatively charged =O groups, like COCH_3_-PhNH_2_^•+^ and SO_3_^−^-PhNH_2_^•+^ (Figure 5a and Table S5).

It is common to introduce explicit H_2_O molecules near the negative and positive charged atoms of the molecules to form hydrogen bonds and improve the calculation accuracy when studying molecules in the aqueous phase [62,63,64]. The purpose of adding H_2_O molecules is to achieve a smooth transition between solute molecules and the implicit solvent depicted by the solvent model. This is also the main reason why placing H_2_O molecules near the (de)ionization groups would improve the accuracy of p*K*_a_ calculations as observed above. However, the improvement of p*K*_a_ calculations by introducing H_2_O molecules at positions beyond the (de)protonation groups of the target molecules has seldom been considered and discussed in previous reports. In this study, an additional H_2_O molecule was further introduced near the =O atom of COCH_3_-PhNH^•^, COCH_3_-PhNH_2_^•+^, SO_3_^−^-PhNH^•^, and SO_3_^−^-PhNH_2_^•+^ to better explore the impact of explicit H_2_O molecules on p*K*_a_ calculations (Figure S5).

Generally, the modifications introduced specifically for these two molecules improved the p*K*_a_ calculation accuracy only when using the M062X(D3)/ma-def2QZVP method (Figure 5b). The RMSE was 0.310 (0.384 before modification), Δp*K*_a M062X_ ranged from 0.003 to 0.685 (average: 0.215), and *r*^2^ was 0.987 (0.977 before modification).

For R-PhNH_3_^+^ (Figure 6a), the CBS-QB3 method yielded a regression line with a slope (*S*) of 1.114 and an intercept (*I*) of −0.356, enabling direct calculations of p*K*_a_. The Δp*K*_a CBS_ value ranged from 0.119 to 1.042 (average: 0.486). By comparison with a previous study (Δp*K*_a CBS_ ranged from 0.42 to 2.90, and the average was 1.52) [20], the results obtained herein were more accurate. The differences in Δp*K*_a CBS_ were mainly influenced by the number of included H_2_O molecules in the models. While the previous study adopted only one H_2_O molecule, we incorporated two H_2_O molecules and significantly improved the accuracy of p*K*_a_ calculations for R-PhNH_3_^+^. These findings further highlight the importance of including H_2_O molecules to increase the accuracy of the p*K*_a_ calculations for R-PhNH_3_^+^ in aqueous solution.

On the other hand, the regression line obtained by the M062X(D3)/ma-def2QZVP method had a slope (*S*) close to 1, indicating a good linear relationship. However, the intercept (*I*) was quite large (*I* = −1.353), deviating notably from the theoretical value of 0, and the Δp*K*_a M062X_ values ranged from 0.922 to 1.856 (average: 1.346). These findings suggest that the errors associated with the M062X(D3)/ma-def2QZVP method appear to be systematic. It is assumed that employing a more accurate method could eliminate the deviations of the intercept (*I*) by increasing all the calculated p*K*_a_ values and shifting the regression line in the positive direction of the y-axis (Figure 6a).

The same strategy of adding extra H_2_O molecules to the substituent groups -COCH_3_ and -SO_3_^−^ was also employed to calculate the p*K*_a_ values of R-PhNH_3_^+^, based on the CBS-QB3 method. Moreover, the M062X(D3)/ma-def2QZVP method was replaced with the more accurate double-hybrid functional method, revDSD-PBEP86-D3(BJ), coupled with the ma-def2QZVPP basis, with the main aim of eliminating systematic errors. The calculated p*K*_a_ values of R-PhNH_3_^+^ after incorporating these modifications are summarized in Table S7 and Figure S6.

For the p*K*_a_ values obtained by the CBS-QB3 method, the addition of H_2_O near the -COCH_3_ and -SO_3_^−^ groups failed to notably enhance, and in the case of -SO_3_^−^ it even decreased the accuracy of calculations (RMSE increased from 0.349 to 0.371, and *r*^2^ of the linear fitting decreased from 0.963 to 0.865). These results suggest that adding extra H_2_O molecules may not always improve p*K*_a_ calculations, as observed in previous studies [65]. On the other hand, the revDSD-PBEP86-D3(BJ)/ma-def2QZVPP method significantly increased the value of the intercept (*I*) from −1.353 to 0.521, while the slope remained close to 1 (*S* = 1.029), which suggests that systematic errors were minimized in this way. Additionally, the strategy of adding extra H_2_O molecules near the R group was further adopted when using the revDSD-PBEP86-D3(BJ)/ma-def2QZVPP method. H_2_O molecules were added near R groups that could form hydrogen bonds with H_2_O, such as negative O atoms (-COCH_3_, -SO_3_^−^, and -OCH_3_), halogen atoms (F and I), and positively charged H in -NH_2_. The calculated p*K*_a_ values are summarized in Table S7, and the modified regression line is shown in Figure 6b. The linear regression analysis yielded a line with *S* = 0.935, *I* = 0.525, and *r*^2^ = 0.884, with an average Δp*K*_a_ value of 0.505. However, the p*K*_a_ of one compound (-SO_3_^−^) deviated significantly from the regression line, with a Δp*K*_a_ value of 1.351. The addition of H_2_O even decreased the calculation accuracy of SO_3_^−^-PhNH_3_^+^. These findings suggest that the revDSD-PBEP86-D3(BJ)/ma-def2QZVPP method may not be suitable for R groups with weak polarity or in anionic form. By excluding SO_3_^−^-PhNH_3_^+^, the calculation accuracy was significantly improved, with Δp*K*_a_ values in the range of 0.089–0.651 (average of 0.247) and a regression line with *S* = 0.986, *I* = 0.202, and *r*^2^ = 0.959.

Although the adoption of revDSD-PBEP86-D3(BJ)/ma-def2QZVPP and extra explicit water molecules near R would improve the p*K*_a_ calculation for most R-PhNH_3_^+^ compounds, this method is computationally quite expensive and time-consuming. Compared with M062X(D3)/ma-def2QZVP (H-PhNH_3_^+^ as a typical example, 501 min), the calculation time for this alternative method was notably longer (1404 min, as illustrated in Figure S4b). Therefore, unless necessary, such as when dealing with molecules containing heavy atoms, we do not recommend revDSD-PBEP86-D3(BJ)/ma-def2QZVPP for p*K*_a_ calculations of compounds with high molecular weight.

## 3. Methods

Quantum chemical calculations were carried out with a computer equipped with 2 AMD EPYC 7R32 CPUs (96 cores, 192 threads total across dual CPUs, maximum clock speed of 3.3 GHz), Gigabyte MZ72-HBO motherboard with integrated graphics card, and sixteen Micron DDR4 3200 RECC random access memory cards (32 GB each).

### 3.1. Calculation of ΔG_deprot_(sol)

#### 3.1.1. Direct Approach

(1) Methods and basis sets

The free energies of the deprotonated and protonated states of molecules were directly calculated based on the SMD model, and Δ*G*_deprot_(sol) was determined according to Equation (2) [15,16].

(2) $$\Delta G_{\mathrm{deprot}}(\mathrm{sol})=G_{A-}(\mathrm{sol})-G_{\mathrm{HA}}(\mathrm{sol})+\Delta G_{H+}(\mathrm{sol})$$

where *G*_A−_(sol) and *G*_HA_(sol) refer to the free energies in water of the deprotonated (A^−^) and protonated states (HA) of the studied compounds, respectively. Δ*G*_H+_(sol) is the free energy of H^+^, which has been experimentally determined as −1131.44 kJ mol^−1^ [4].

(2) Corrections of G(sol)

Corrections of *G*(sol) were obtained based on scale factors for the Zero Point Energy (ZPE) [66], taking into account the harmonic approximation. All corrections were performed using the Shermo program (version: 2.3.5) [67]. The program incorporates three models of harmonic approximation, including the rigid-rotor harmonic oscillator (RRHO) method (labeled as C0) and two quasi-RRHO (QRRHO) methods. The first QRRHO method (QRRHO1, labeled as C1) was suggested by Truhlar, artificially raising all frequencies lower than 100 cm^−1^ to 100 cm^−1^ [68]. The second QRRHO method (QRRHO2, labeled as C2) was suggested by Grimme and was based on an interpolation method [67,69].

#### 3.1.2. Indirect (Thermodynamic) Approach

(1) Methods and basis sets

The indirect approach is based on thermodynamic cycles for calculating the value of *G*(sol) (Equation (3)) [15,16].

(3) G(sol)=G(gas)+ΔG(sol)

where *G*(gas) and Δ*G*(sol) represent, respectively, gas-phase free energy and solvation free energy of a specific compound. These two values were calculated separately using different methods and basis sets. Specifically, for compounds containing heavy atoms, *G*(gas) was calculated using the M062X(D3)/ma-def2QZVP method [16,52,57,58]. Additionally, Δ*G*(sol) was calculated based on six different protocols, with and without explicit water molecules (Table 2). Then, by combining the results of *G*(gas) and Δ*G*(sol), Δ*G*_deprot_(sol) was determined according to Equations (4) and (5).

(4) $$\Delta G_{\mathrm{deprot}}(\mathrm{sol})=\Delta G(\mathrm{gas})+\Delta \Delta G(\mathrm{sol})+\Delta G_{H+}(\mathrm{sol})$$

(5) $$\Delta G_{\mathrm{deprot}}(\mathrm{sol})=[G_{A-}(\mathrm{gas})-G_{\mathrm{HA}}(\mathrm{gas})]+[\Delta G_{A-}(\mathrm{sol})-\Delta G_{\mathrm{HA}}(\mathrm{sol})]+\Delta G_{H+}(\mathrm{sol})$$

(2) Corrections of *G*(gas)

Corrections to the values of *G*(gas) were carried out with Shermo software (version: 2.3.5), as mentioned above [48,49,50].

#### 3.1.3. Data Processing and Advanced Modification Method

The root mean square error (RMSE, calculated according to Equation (6) where *n* indicates the total number of calculated molecules) and linear regression analysis were basically used as standards to select and improve appropriate models and combinations of calculation methods/parameters. If the attempts to achieve higher accuracy using CBS-QB3 and M062X(D3)/ma-def2QZVP methods did not succeed (RMSE exceeded 1 or the correlation coefficient *r*^2^ < 0.9), the doubly hybrid functional revDSD-PBEP86-D3(BJ) in conjunction with ma-def2QZVPP was further employed to improve the accuracy of the calculations [57,58,72].

(6) $$\mathrm{RMSE}=\sqrt{\frac{{{(\mathrm{pKa}}_{\mathrm{Calculation}}-{\mathrm{pKa}}_{\mathrm{Experiment}})}^{2}}{n}}$$

## 4. Conclusions

In this study, we constructed various molecular models by altering the number and positions of H_2_O molecules towards p*K*_a_ calculations for R-PhNH_2_^•+^ and R-PhNH_3_^+^ in the aqueous phase. Then, based on the appropriate models, we evaluated the performance of direct and indirect approaches, CBS-QB3 and M062X(D3)/ma-def2QZVP methods, along with methods for correcting free energy and calculating solvation energy.

For R-PhNH_2_^•+^, both CBS-QB3 and M062X(D3)/ma-def2QZVP produced accurate p*K*_a_ values using models with one or two H_2_O molecules. Additional H_2_O near oxygen-containing substituents further improved the results. CBS-QB3 performed best with the indirect approach, C1 correction method, and ωb97xd/6-31+g(d,p) method for solvation energy calculation, resulting in an average Δp*K*_a_ = 0.376. M062X(D3)/ma-def2QZVP performed best with the direct approach and C1 correction method, yielding an average Δp*K*_a_ = 0.215. These methods highlight a potential to predict p*K*_a_ values of other environmentally important radical cations, such as protonated phenoxyl radicals.

Predicting p*K*_a_ values for R-PhNH_3_^+^ was more challenging compared to R-PhNH_2_^•+^. CBS-QB3 resulted in an average Δp*K*_a_ = 0.486 with two H_2_O molecules in the model, while this method was useless for molecules with heavy atoms. M062X(D3)/ma-def2QZVP failed to accurately calculate p*K*_a_ values, with an average Δp*K*_a_ = 1.351 based on models with three H_2_O molecules. Although the adoption of the revDSD-PBEP86-D3/ma-def2QZVPP method and the inclusion of extra H_2_O near the R group improved the results (average Δp*K*_a_ = 0.357), it is worth noting that the computational time requirements limit the method’s application when dealing with compounds having high molecular weight.

## Figures and Tables

**Table 1:** The reported p*K*_a_ values of R-PhNH_2_^•+^ and R-PhNH_3_^+^.

R		H	C_4_H_9_	CF_3_	CH_3_	OCH_3_	CN	COCH_3_	I	NH_2_	SO_3_^−^
p*K*_a_	R-PhNH_2_^•+^	7.05 [35]	8.2 [7]	4.8 [7]	8.5 [36]	9.6 [7]	4 [7]	6.1 [7]	7.1 [7]	12 [7]	5.8 [7]
	R-PhNH_3_^+ a^	4.62 [37], 4.58 [38]	4.95 [34]	2.92 [39], 2.75 [40], 2.57 [41]	5.12 [37]	5.29 [37]	1.75 [42], 1.82 [43]	2.19 [42], 2.26 [43]	3.78 [44]	5.94 [45], 6.2 [42]	3.25 [46], 2.93 [47], 3.32 [48]

^a^: Average p*K*_a_ values were adopted if more than two values were available.

**Table 2:** Protocols for calculating ΔΔ*G*_HA_(sol) (Δ*G*_A−_(sol) − Δ*G*_HA_(sol)), with and without explicit water molecules.

No.	Models	Methods ^a^	Ref.
P1 ^b^	Implicit (single)	M052X/6-31G(d)	[14]
P2 ^c^	Implicit (mixed)	M052X/6-31G(d) for neutral, e.g., R-PhNH^•^ and R-PhNH_2_ M052X/6-31+G(d,p) for cation, e.g., R-PhNH_2_^•+^ and R-PhNH_3_^+^ HF/6-31G(d) for anion, e.g., SO_3_^−^-PhNH^•^ and SO_3_^−^-PhNH_2_ ^d^ HF/6-31G(d) for SO_3_^−^-PhNH^•+^ and SO_3_^−^-PhNH_3_^+^	[14]
P3 ^e^	Implicit (mixed)	M052X/6-31G(d) for radical cation, e.g., R-PhNH_2_^•+^ HF/6-31G(d) for neutral and anion radical, e.g., R-PhNH^•^ ^d^ HF/6-31G(d) for SO_3_^−^-PhNH^•+^	-
P4	Explicit	M062X/6-31G(d)	[70]
P5	Explicit	M052X/cc-pVTZ	[71]
P6	Explicit	ωb97xd/6-31+g(d,p)	[17]

^a^: Compounds containing iodine atoms were calculated based on the ma-def2QZVP basis set [57,58]. ^b^: M052X/6-31G(d) was the best overall average performer for aqueous solvation energies [14]. ^c^: M052X/6-31G(d), M052X/6-31+G(d,p), and HF/6-31G(d) were the best for neutrals, cations, and anions, respectively [14]. ^d^: Since -SO_3_^−^ carries a negative charge, molecules in neutral and cationic form containing this group were also calculated using HF/6-31G(d), which worked best for anions [14]. Finally, the closest value to the experimental pK_a_ was selected. ^e^: As R-PhNH_2_^•+^ is not a typically stable cation as those parameterized in the original reference [14], Protocol-3 was introduced as an imitation of Protocol-2 (P2). R-PhNH_2_^•+^ and R-PhNH^•^ were calculated with M052X/6-31G(d) and HF/6-31G(d), respectively.

## Data Availability

Data are contained within the article or Supplementary Materials.

## Supplementary Materials

The following supporting information can be downloaded at: https://www.mdpi.com/article/10.3390/molecules29194522/s1, Table S1: The calculated energies of R-PhNH^•^ (A^−^) and R-PhNH_2_^•+^ (AH) based on direct method (unit: a.u.); Table S2: The calculated energies of R-PhNH^•^ (A^−^) and R-PhNH_2_^•+^ (AH) based on indirect method (unit: a.u.); Table S3: The calculated energies of R-PhNH_2_ (A^−^) and R-PhNH_3_^+^ (AH) based on direct method (unit: a.u.); Table S4: The calculated energies of R-PhNH_2_ (A^−^) and R-PhNH_3_^+^ (AH) based on indirect method (unit: a.u.); Table S5: The experimental and calculated p*K*_a_ values of PhNH_2_^•+^; Table S6: The experimental and calculated p*K*_a_ values of PhNH_3_^+^; Table S7: p*K*_a_ values of R-PhNH_3_^+^ calculated by revDSD-PBEP86-D3(BJ)/ma-def2QZVPP with models included three H_2_O combined with amino group; Figure S1: The potential positions of H_2_O molecules in the models. H-PhNH^•^, H-PhNH_2_^•+^, H-PhNH_2_ and H-PhNH_3_^+^ are presented as examples; Figure S2: Comparison of H-PhNH_2_ models with three H_2_O molecules optimized by M062X(D3)/6-311++g(d,p) and calculated based on CBS-QB3; Figure S3: The changes in molecular structures after the calculation of *G*(gas) and *G*(sol) based on the CBS-QB3 method; Figure S4: The calculation time for (a) H-PhNH_2_^•+^ (indirect approach/CBS-QB3/C1/P6/1H2O and direct approach/M062X(D3)/ma-def2QZVP/C1/2H2O), and (b) H-PhNH_3_^+^ (direct approach/CBS-QB3/C1/2H2O, direct approach/M062X(D3)/ma-def2QZVP/C1/3H2O, and direct approach/M062X(D3)/revDSD-PBEP86-D3(BJ)/ma-def2QZVPP/3H2O). The number of processors is 20; Figure S5: The optimized structures of (a) COCH_3_-PhNH^•^, (b) COCH_3_-PhNH_2_^•+^, (c) SO_3_^−^-PhNH^•^ and (d) SO_3_^−^-PhNH_2_^•+^ with an additional H_2_O near =O; Figure S6: The performance of modified CBS-QB3 and revDSD-PBEP86-D3(BJ)/ma-def2QZVPP methods on pKa calculations of R-PhNH_3_^+^.
