# Supplementary material for: Predicting pKa Values of Para-Substituted Aniline Radical Cations vs. Stable Anilinium Ions in Aqueous Media
Source: Molecules. 2024 Sep 24;29(19):4522. doi: 10.3390/molecules29194522 (PMC11477995; doi:10.3390/molecules29194522)
Supplement: Supplementary file 1 [file molecules-29-04522-s001.zip › molecules-3120349-supplementary.pdf]

Predicting pK_a values of para-substituted aniline radical cations vs. stable anilinium ions in aqueous media

Jingxin Wang [1], Hansun Fang [2,*], Zixi Zhong [5], Huajun Huang [2], Ximei Liang [3], Yufan Yuan [2], Wenwen Zhou [2], Davide Vione [4,*]

[1] Guangdong Provincial Engineering Technology Research Center of Public Health Detection and Assessment, School of Public Health, Guangdong Pharmaceutical University, Guangzhou 510310, China

[2] Key Laboratory of Poyang Lake Basin Agricultural Resource and Ecology of Jiangxi Province, College of Land Resource and Environment, Jiangxi Agricultural University, Nanchang 330045, China

[3] College of Animal Science and Technology, Jiangxi Agricultural University, Nanchang 330045, China

[4] Dipartimento di Chimica, Università di Torino, Via P. Giuria 5, 10125 Torino, Italy.

[5] Guangzhou Foreign Language School, Guangzhou 511455, China

* Corresponding author: e-mail: davide.vione@unito.it (D. Vione); fanghansun@163.com (H.S. Fang)

Number of pages: 36

		Pages
Table S1	The calculated energies of R-PhNH$^{\bullet}$ (A^{-}) and R-$PhNH_2^{\bullet+}$ (AH) based on direct method (unit: a.u.).	S3
Table S2	The calculated energies of R-PhNH$^{\bullet}$ (A^{-}) and R-$PhNH_2^{\bullet+}$ (AH) based on indirect method (unit: a.u.)	S8
Table S3	The calculated energies of R-$PhNH_2$ (A^{-}) and R-$PhNH_3^{+}$ (AH) based on direct method (unit: a.u.).	S13
Table S4	.The calculated energies of R-$PhNH_2$ (A^{-}) and R-$PhNH_3^{+}$ (AH) based on indirect method (unit: a.u.).	S18
Table S5	The experimental and calculated pK_a values of $PhNH_2^{\bullet+}$.	S23
Table S6	The experimental and calculated pK_a values of $PhNH_3^{+}$.	S28
Table S7	pK_a values of R-$PhNH_3^{+}$ calculated by revDSD-PBEP86-D3(BJ)/ma-def2QZVPP with models including three H_2O combined with amino group.	S33
Figure S1	The potential positions of H_2O molecules in the models. H-PhNH$^{\bullet}$, H-$PhNH_2^{\bullet+}$, H-$PhNH_2$ and H-$PhNH_3^{+}$ are presented as examples.	S34
Figure S2	Comparison of H-$PhNH_2$ models with three H_2O molecules optimized by M062X(D3)/6-311++g(d,p) and calculated based on CBS-QB3.	S34
Figure S3	The changes in molecular structures after the calculation of G(gas) and G(sol) based on the CBS-QB3 method.	S35
Figure S4	The calculation time for (a) H-$PhNH_2^{\bullet+}$ (indirect approach/CBS-QB3/C1/P6/1H_2O and direct approach/M062X(D3)/ma-def2QZVP/C1/2H_2O), and (b) H-$PhNH_3^{+}$ (direct approach/CBS-QB3/C1/2H_2O, direct approach/M062X(D3)/ma-def2QZVP/C1/3H_2O, and direct approach/M062X(D3)/revDSD-PBEP86-D3(BJ)/ma-def2QZVPP/3H_2O). The number of processors is 20.	S35
Figure S5	The optimized structures of (a) $COCH_3$-PhNH$^{\bullet}$, (b) $COCH_3$-$PhNH_2^{\bullet+}$, (c) SO_3^{-}-PhNH$^{\bullet}$ and (d) SO_3^{-}-$PhNH_2^{\bullet+}$ with an additional H_2O near =O.	S36
Figure S6	The performance of modified CBS-QB3 and revDSD-PBEP86-D3(BJ)/ma-def2QZVPP methods on pK_a calculations of R-$PhNH_3^{+}$.	S36

Table S1. The calculated energies of R-PhNH$^{•}$ (A^{-}) and R-$PhNH_2^{•+}$ (AH) based on direct method (unit: a.u.).

R	Calculation method	Number of H2O	Models of A^{-}-HA	A-			AH		
				C0	C1	C2	C0	C1	C2
C_4H_9	CBS-QB3	0	/	-443.3554638	-443.3549046	-443.354756	-443.8037798	-443.8028526	-443.8027795
CF_3				-623.1943550	-623.1928095	-623.1930623	-623.6349494	-623.6329322	-623.6333760
CH_3O				-400.8036630	-400.8035705	-400.8034437	-401.2549827	-401.2549085	-401.2547875
CH_3				-325.6716611	-325.6705553	-325.6707132	-326.1198038	-326.1188345	-326.1188393
CN				-378.5653023	-378.5652844	-378.5651427	-379.0051706	-379.0048226	-379.0047414
$COCH_3$				-438.8672020	-438.8664842	-438.8663809	-439.3090434	-439.3077512	-439.3078689
NH_2				-341.7297024	-341.7297024	-341.7296643	-342.1905455	-342.1905084	-342.1903859
H				-286.4364216	-286.4364216	-286.4364256	-286.8821474	-286.8821474	-286.8821391
SO_3^{-}				-909.2380783	-909.2360343	-909.2365081	-909.6826079	-909.6804528	-909.6809389
				C0	C1	C2	C0	C1	C2
C_4H_9	CBS-QB3	1	*HO*-HO	-519.7124278	-519.7090739	-519.7093516	-520.1608010	-520.1588245	-520.1586683
CF_3				-699.5503064	-699.5466368	-699.5471048	-699.9924398	-699.9893799	-699.9897687
CH_3O				-477.1598453	-477.1576263	-477.1576286	-477.6121476	-477.6110925	-477.6108451
CH_3				-402.0258727	-402.0241894	-402.0239119	-402.4757996	-402.4745546	-402.4742436
CN				-454.9203961	-454.9191870	-454.9189308	-455.3621854	-455.3613129	-455.3609572
$COCH_3$				-515.2220680	-515.2201398	-515.2199414	-515.6661217	-515.6642194	-515.6640606
NH_2				-418.0848631	-418.0836195	-418.0833395	-418.5461028	-418.5453775	-418.5451308
H				-362.7905249	-362.7896370	-362.7894639	-363.2386844	-363.2380324	-363.2377462
SO_3^{-}				-985.5926906	-985.5900013	-985.5899884	-986.0396593	-986.0368765	-986.0370520
				C0	C1	C2	C0	C1	C2
C_4H_9	CBS-QB3	1	*NH*-HO	-519.7154872	-519.7131802	-519.7131303	-520.1608010	-520.1588245	-520.1586683
CF_3				-699.5554800	-699.5504042	-699.5515812	-699.9924398	-699.9893799	-699.9897687
CH_3O				-477.1635907	-477.1619497	-477.1619118	-477.6121476	-477.6110925	-477.6108451

CH_3				-402.0312415	-402.0288156	-402.0288156	-402.4757996	-402.4745546	-402.4742436
CN				-454.9241583	-454.9223610	-454.9223889	-455.3621854	-455.3613129	-455.3609572
$COCH_3$				-515.2264058	-515.2239923	-515.2240036	-515.6661217	-515.6642194	-515.6640606
NH_2				-418.0912106	-418.0895600	-418.0894606	-418.5461028	-418.5453775	-418.5451308
H				-362.7958985	-362.7943299	-362.7944149	-363.2386844	-363.2380324	-363.2377462
SO_3^-				-985.5968164	-985.5939402	-985.5941365	-986.0396593	-986.0368765	-986.0370520
				C0	C1	C2	C0	C1	C2
C_4H_9	CBS-QB3	2	*NHHO*-2HO	-596.0718333	-596.0673489	-596.0674374	-596.5180067	-596.5151435	-596.5147147
CF_3				-775.9095445	-775.9044862	-775.9048540	-776.3490995	-776.3460818	-776.3458457
CH_3O				-553.5199681	-553.5163303	-553.5163423	-553.9695361	-553.9673606	-553.9668706
CH_3				-478.3879572	-478.3829688	-478.3833599	-478.8339088	-478.8308556	-478.8306312
CN				-531.2799194	-531.2767192	-531.2765626	-531.7193010	-531.7177539	-531.7171718
$COCH_3$				-591.5832111	-591.5782773	-591.5785442	-592.0244479	-592.0209460	-592.0208596
NH_2				-494.4467112	-494.4434136	-494.4432755	-494.9037500	-494.9013712	-494.9010268
H				-439.1506914	-439.1481752	-439.1479525	-439.5958621	-439.5943384	-439.5938112
SO_3^-				-1061.9528320	-1061.9482738	-1061.9483357	-1062.3965026	-1062.3933918	-1062.3930990
				C0	C1	C2	C0	C1	C2
C_4H_9	M062X/def2QZVP	0	/	-444.0094359	-444.0088767	-444.0087281	-444.4590879	-444.4581607	-444.4580876
CF_3				-624.0052513	-624.0037058	-624.0039586	-624.4451877	-624.4431705	-624.4436143
CH_3O				-401.4037705	-401.4036781	-401.4035513	-401.8568782	-401.856804	-401.8566829
CH_3				-326.1841871	-326.1830813	-326.1832392	-326.6338456	-326.6328763	-326.632881
CN				-379.1530847	-379.1530668	-379.152925	-379.5914284	-379.5910804	-379.5909992
$COCH_3$				-439.5248299	-439.5241121	-439.5240087	-439.966407	-439.9651148	-439.9652325
I				-583.9394577	-583.9393919	-583.9392757	-584.3858004	-584.3853885	-584.3853383
NH_2				-342.2583816	-342.2583816	-342.2583435	-342.7194543	-342.7194171	-342.7192946
H				-286.8911859	-286.8911859	-286.8911899	-287.3367089	-287.3367089	-287.3367005

SO_3^-				-910.3881903	-910.3861462	-910.38662	-910.831854	-910.829699	-910.830185
				C0	C1	C2	C0	C1	C2
C_4H_9	M062X/def2QZVP	1	*HO*-HO	-520.4745866	-520.4712327	-520.4715104	-520.9244547	-520.9224783	-520.922322
CF_3				-700.4394365	-700.4357668	-700.4362348	-700.8811542	-700.8780942	-700.878483
CH_3O				-477.8382715	-477.8360524	-477.8360548	-478.2925526	-478.2914975	-478.2912501
CH_3				-402.6169195	-402.6152361	-402.6149586	-403.0684401	-403.0671951	-403.0668841
CN				-455.5864388	-455.5852297	-455.5849735	-456.0277375	-456.026865	-456.0265093
$COCH_3$				-515.9579497	-515.9560215	-515.9558231	-516.401982	-516.4000797	-516.3999209
I				-660.3724049	-660.3711567	-660.3708997	-660.8214111	-660.8203513	-660.8200348
NH_2				-418.691852	-418.6906084	-418.6903284	-419.1546892	-419.1539639	-419.1537172
H				-363.3238943	-363.3230064	-363.3228333	-363.7726869	-363.7720348	-363.7717487
SO_3^-				-986.8208522	-986.8181629	-986.81815	-987.2673402	-987.2645574	-987.2647329
				C0	C1	C2	C0	C1	C2
C_4H_9	M062X/def2QZVP	1	*NH*-HO	-520.4780263	-520.4757193	-520.4756694	-520.9244547	-520.9224783	-520.922322
CF_3				-700.4448003	-700.4397246	-700.4409015	-700.8811542	-700.8780942	-700.878483
CH_3O				-477.8425064	-477.8408654	-477.8408274	-478.2925526	-478.2914975	-478.2912501
CH_3				-402.6226751	-402.620004	-402.6202493	-403.0684401	-403.0671951	-403.0668841
CN				-455.590507	-455.5887098	-455.5887377	-456.0277375	-456.026865	-456.0265093
$COCH_3$				-515.9625008	-515.9600873	-515.9600986	-516.401982	-516.4000797	-516.3999209
I				-660.3773748	-660.3757622	-660.3756679	-660.8214111	-660.8203513	-660.8200348
NH_2				-418.699308	-418.6976574	-418.697558	-419.1546892	-419.1539639	-419.1537172
H				-363.3293535	-363.327785	-363.3278699	-363.7726869	-363.7720348	-363.7717487
SO_3^-				-986.8248697	-986.8219935	-986.8221898	-987.2673402	-987.2645574	-987.2647329
				C0	C1	C2	C0	C1	C2
C_4H_9	M062X/def2QZVP	2	*NHHO*-2HO	-596.9123881	-596.9079038	-596.9079922	-597.35982	-597.3569568	-597.3565279
CF_3				-776.8769215	-776.8718632	-776.872231	-777.3162678	-777.3132501	-777.313014

CH_3O				-554.2770651	-554.2734274	-554.2734393	-554.7281978	-554.7260223	-554.7255323
CH_3				-479.0576275	-479.0526391	-479.0530302	-479.504961	-479.5019077	-479.5016833
CN				-532.0245506	-532.0213504	-532.0211937	-532.4627721	-532.4612249	-532.4606428
$COCH_3$				-592.3974805	-592.3925467	-592.3928136	-592.8387028	-592.8352009	-592.8351145
I				-736.8114193	-736.8080572	-736.8079623	-737.256254	-737.2542294	-737.2537264
NH_2				-495.1329298	-495.1296322	-495.1294941	-495.5899914	-495.5876126	-495.5872681
H				-439.7627642	-439.760248	-439.7600253	-440.2078437	-440.20632	-440.2057928
SO_3^-				-1063.259019	-1063.254461	-1063.254523	-1063.70258	-1063.699469	-1063.699176
				C0	C1	C2	C0	C1	C2
C_4H_9	M062X/def2QZVP	2	*NHA90*-HOA90	-596.9162013	-596.9122061	-596.9121918	-597.3599448	-597.3561135	-597.356078
CF_3				-776.8791876	-776.874667	-776.8748368	-777.3170457	-777.3117746	-777.312219
CH_3O				-554.2816355	-554.2779497	-554.2778649	-554.7280534	-554.7253287	-554.7249384
CH_3				-479.0606248	-479.0567458	-479.0566328	-479.5037449	-479.5009121	-479.5005364
CN				-532.0277169	-532.0237979	-532.0238607	-532.4620554	-532.4596818	-532.4594241
$COCH_3$				-592.4005993	-592.3962547	-592.3962127	-592.8357539	-592.8325497	-592.8324472
I				-736.8160539	-736.8120218	-736.8121954	-737.256696	-737.2536939	-737.2534085
NH_2				-495.1374389	-495.1341793	-495.13404	-495.5916864	-495.5892457	-495.5888189
H				-439.7683812	-439.7646683	-439.7647221	-440.2079627	-440.2057316	-440.2053594
SO_3^-				-1063.262144	-1063.258037	-1063.25823	-1063.703595	-1063.699253	-1063.699688
				C0	C1	C2	C0	C1	C2
C_4H_9	M062X/def2QZVP	2	*HOA90*-HOA90	-596.9104811	-596.9073124	-596.9069165	-597.3599448	-597.3561135	-597.356078
CF_3				-776.8768503	-776.8717164	-776.8719992	-777.3170457	-777.3117746	-777.312219
CH_3O				-554.2747663	-554.2721079	-554.271658	-554.7280534	-554.7253287	-554.7249384
CH_3				-479.0550832	-479.0517947	-479.0513886	-479.5037449	-479.5009121	-479.5005364
CN				-532.0234623	-532.0209232	-532.0204921	-532.4620554	-532.4596818	-532.4594241
$COCH_3$				-592.3960637	-592.3923408	-592.3920365	-592.8357539	-592.8325497	-592.8324472

I				-736.8102257	-736.8074184	-736.8070783	-737.256696	-737.2536939	-737.2534085
NH_2				-495.1287057	-495.126663	-495.1262924	-495.5916864	-495.5892457	-495.5888189
H				-439.7626041	-439.7598604	-439.7596536	-440.2079627	-440.2057316	-440.2053594
SO_3^-				-1063.257955	-1063.254262	-1063.254255	-1063.703595	-1063.699253	-1063.699688
				C0	C1	C2	C0	C1	C2
C_4H_9				-673.3487673	-673.3438109	-673.3436085	-673.7949514	-673.7912951	-673.7906858
CF_3				-853.3132679	-853.3070669	-853.307209	-853.7521998	-853.7465804	-853.7466107
CH_3O				-630.7151738	-630.7097016	-630.7096977	-631.1642556	-631.1598532	-631.15954
CH_3				-555.4927114	-555.4878251	-555.4876961	-555.9399693	-555.9361083	-555.9355406
CN	M062X/def2QZVP	3	*NHHOA90*-2HOA90	-608.4611798	-608.4564757	-608.4562355	-608.8983539	-608.8945566	-608.8942724
$COCH_3$				-668.8340011	-668.8280596	-668.8280462	-669.2729379	-669.2681729	-669.2679042
I				-813.2495803	-813.2439261	-813.2440854	-813.6917947	-813.6881088	-813.6875434
NH_2				-571.5701049	-571.5657665	-571.5655006	-572.0273752	-572.0221332	-572.0223134
H				-516.2004238	-516.1960803	-516.1958998	-516.6433956	-516.6403935	-516.6399123
SO_3^-				-1139.695738	-1139.689775	-1139.690143	-1140.138481	-1140.13398	-1140.133881
				C0	C1	C2	C0	C1	C2
C_4H_9				-749.788593	-749.7805969	-749.7807852	-750.2312526	-750.2257907	-750.2251891
CF_3				-929.7486232	-929.7414465	-929.7413549	-930.1865409	-930.1795807	-930.1794602
CH_3O				-707.1503134	-707.144124	-707.144144	-707.5969628	-707.5921232	-707.5916943
CH_3				-631.9317958	-631.9242901	-631.9243077	-632.3776035	-632.3710493	-632.37064
CN	M062X/def2QZVP	4	*NHHO2A90*-2HO2A90	-684.8965868	-684.890711	-684.8902233	-685.3345307	-685.3287219	-685.3282259
$COCH_3$				-745.2721039	-745.2637208	-745.2638253	-745.7091659	-745.7022222	-745.7019929
I				-889.6885781	-889.6806528	-889.6806509	-890.1284661	-890.1219452	-890.1218299
NH_2				-648.0076516	-648.0017199	-648.0015959	-648.4644736	-648.4588435	-648.4583123
H				-592.6385151	-592.631962	-592.631908	-593.079404	-593.0744571	-593.0737092
SO_3^-				-1216.133242	-1216.125859	-1216.126018	-1216.573867	-1216.567408	-1216.567395

Table S2. The calculated energies of R-PhNH$^{\bullet}$ (A^-) and R-PhNH$_2^{\bullet+}$ (AH) based on indirect method (unit: a.u.).

R	Calculation method	Number of H_2O	Models of A^--HA	A^-			AH			Solvation energy		
				C0	C1	C2	C0	C1	C2	P1	P2	P3
C_4H_9	CBS-QB3	0	/	−443.3477595	−443.3472003	−443.3470517	−443.7186632	−443.717736	−443.7176629	0.075370068	0.076875036	0.076431968
CF_3				−623.1872083	−623.1856628	−623.1859156	−623.5346439	−623.5326267	−623.5330705	0.090355932	0.093761246	0.089749822
CH_3O				−400.7927281	−400.7926356	−400.7925088	−401.1692985	−401.1692243	−401.1691033	0.073443271	0.07529814	0.074063195
CH_3				−325.6627228	−325.6616170	−325.6617749	−326.0305615	−326.0295922	−326.0295970	0.078697922	0.079957415	0.079985822
CN				−378.5537794	−378.5537615	−378.5536198	−378.8985307	−378.8981827	−378.8981015	0.092455261	0.094830688	0.09166268
$COCH_3$				−438.8543158	−438.8535980	−438.8534947	−439.2096134	−439.2083212	−439.2084389	0.084659962	0.08757362	0.083460046
NH_2				−341.7122634	−341.7122634	−341.7122253	−342.1001041	−342.1000670	−342.0999445	0.070933167	0.072622631	0.074846279
H				−286.4276809	−286.4276809	−286.4276849	−286.7882006	−286.7882006	−286.7881923	0.083181754	0.084463963	0.084050505
SO_3^-				−909.1363447	−909.1343007	−909.1347745	−909.6056205	−909.6034654	−909.6039515	−0.03043927	−0.037478617	−0.037478617
				C0	C1	C2	C0	C1	C2	P4	P5	P6
C_4H_9	CBS-QB3	1	HO-HO	−519.6969218	−519.6935679	−519.6938456	−520.0813382	−520.0793617	−520.0792055	0.060796694	0.061423636	0.061451078
CF_3				−699.536749	−699.5330794	−699.5335474	−699.9002340	−699.8971741	−699.8975629	0.074550364	0.07675384	0.076450798
CH_3O				−477.1410492	−477.1388302	−477.1388325	−477.5319759	−477.5309208	−477.5306734	0.058688908	0.059826998	0.059450179
CH_3				−402.0094532	−402.0077699	−402.0074924	−402.3928899	−402.3916449	−402.3913339	0.063372054	0.064246663	0.064009694
CN				−454.9024858	−454.9012767	−454.9010205	−455.2634271	−455.2625546	−455.2621989	0.077057003	0.079120363	0.078657184
$COCH_3$				−515.2021617	−515.2002335	−515.2000351	−515.5736239	−515.5717216	−515.5715628	0.069331593	0.070911576	0.070789341
NH_2				−418.0592082	−418.0579646	−418.0576846	−418.4604395	−418.4597142	−418.4594675	0.056266151	0.057542082	0.057293271
H				−362.7742817	−362.7733938	−362.7732207	−363.1520187	−363.1513667	−363.1510805	0.06709095	0.068298106	0.068021219
SO_3^-				−985.4780419	−985.4753526	−985.4753397	−985.9603654	−985.9575826	−985.9577581	−0.04283444	−0.039429498	−0.038350523
				C0	C1	C2	C0	C1	C2	P4	P5	P6
C_4H_9	CBS-QB3	1	NH	−519.7001831	−519.6978761	−519.6978262	−520.0813382	−520.0793617	−520.0792055	0.060173819	0.061242085	0.060949428

CF_3			-HO	-699.5404521	-699.5353763	-699.5365533	-699.9002340	-699.8971741	-699.8975629	0.072314686	0.074814912	0.074066267
CH_3O				-477.1451728	-477.1435318	-477.1434939	-477.5319759	-477.5309208	-477.5306734	0.058188639	0.059680957	0.058993323
CH_3				-402.0148473	-402.0121761	-402.0124214	-402.3928899	-402.3916449	-402.3913339	0.06254247	0.063835063	0.063275393
CN				-454.9048826	-454.9030853	-454.9031132	-455.2634271	-455.2625546	-455.2621989	0.074703873	0.077101916	0.076307378
$COCH_3$				-515.2061048	-515.2036913	-515.2037026	-515.5736239	-515.5717216	-515.5715628	0.068026151	0.069951473	0.069479622
NH_2				-418.0660456	-418.064395	-418.0642956	-418.4604395	-418.4597142	-418.4594675	0.055821807	0.057343763	0.057028351
H				-362.7790261	-362.7774575	-362.7775425	-363.1520187	-363.1513667	-363.1510805	0.06596643	0.067547164	0.066843733
SO_3^-				-985.4915821	-985.4887059	-985.4889022	-985.9603654	-985.9575826	-985.9577581	-0.03203978	-0.029274256	-0.028925672
				C0	C1	C2	C0	C1	C2	P4	P5	P6
C_4H_9				-596.0496494	-596.0451650	-596.0452535	-596.4425833	-596.4397201	-596.4392913	0.04859489	0.050347679	0.050047837
CF_3				-775.8890397	-775.8839814	-775.8843492	-776.2629181	-776.2599004	-776.2596643	0.059989889	0.063133326	0.062485864
CH_3O				-553.4945050	-553.4908672	-553.4908792	-553.8929572	-553.8907817	-553.8902917	0.04673897	0.048906616	0.048316184
CH_3				-478.3645344	-478.3595460	-478.3599371	-478.7555861	-478.7525329	-478.7523085	0.050499786	0.052499207	0.05196183
CN	CBS-QB3	2	NHHO<br>-2HO	-531.2553101	-531.2521099	-531.2519533	-531.6268332	-531.6252861	-531.6247040	0.062130976	0.06519473	0.064379289
$COCH_3$				-591.5566128	-591.5516790	-591.5519459	-591.9370495	-591.9335476	-591.9334612	0.055875124	0.058483085	0.057970764
NH_2				-494.4142969	-494.4109993	-494.4108612	-494.8215464	-494.8191676	-494.8188232	0.044474966	0.046706693	0.046410862
H				-439.1276184	-439.1251022	-439.1248795	-439.5144091	-439.5128854	-439.5123582	0.053441546	0.055742327	0.0551133
SO_3^-				-1061.8358720	-1061.8313138	-1061.8313757	-1062.3142192	-1062.3111084	-1062.3108156	-0.04139573	-0.03824636	-0.03751406
				C0	C1	C2	C0	C1	C2	P1	P2	P3
C_4H_9				-444.0010828	-444.0005236	-444.000375	-444.375536	-444.3746088	-444.3745357	0.075347213	0.076852181	0.076396002
CF_3				-623.9979345	-623.996389	-623.9966418	-624.3463808	-624.3443637	-624.3448074	0.090355932	0.093761246	0.089749822
CH_3O	M062X/<br>def2QZVP	0	/	-401.3918965	-401.391804	-401.3916772	-401.7715026	-401.7714284	-401.7713073	0.073443271	0.07529814	0.074063195
CH_3				-326.1748019	-326.173696	-326.173854	-326.5457923	-326.544823	-326.5448277	0.078697922	0.079957415	0.079985822
CN				-379.140915	-379.1408971	-379.1407554	-379.4862959	-379.4859479	-379.4858667	0.092455261	0.094830688	0.09166268
$COCH_3$				-439.5105216	-439.5098038	-439.5097004	-439.8671423	-439.8658501	-439.8659678	0.084659962	0.08757362	0.083460046

I				−583.9288879	−583.9288222	−583.928706	−584.2937831	−584.2933713	−584.293321	0.082816447	0.083083557	0.082544148
NH_2				−342.239081	−342.239081	−342.2390429	−342.6290664	−342.6290292	−342.6289067	0.070933167	0.072622631	0.074846279
H				−286.8819379	−286.8819379	−286.8819419	−287.2438779	−287.2438779	−287.2438695	0.083181754	0.084463963	0.084050505
SO_3^-				−910.2842303	−910.2821863	−910.2826601	−910.7553984	−910.7532434	−910.7537294	−0.03043927	−0.037478617	−0.037478617
				C0	C1	C2	C0	C1	C2	P4	P5	P6
C_4H_9	M062X/ def2QZVP	1	HO -HO	−520.4583314	−520.4549775	−520.4552552	−520.8462525	−520.844276	−520.8441198	0.060796694	0.061423636	0.061451078
CF_3				−700.4256999	−700.4220302	−700.4224983	−700.7902076	−700.7871476	−700.7875364	0.074550364	0.07675384	0.076450798
CH_3O				−477.8184648	−477.8162457	−477.8162481	−478.2124824	−478.2114273	−478.2111799	0.058688908	0.059826998	0.059450179
CH_3				−402.5997179	−402.5980346	−402.5977571	−402.9864482	−402.9852032	−402.9848921	0.063372054	0.064246663	0.064009694
CN				−455.568152	−455.5669428	−455.5666867	−455.9302225	−455.92935	−455.9289943	0.077057003	0.079120363	0.078657184
$COCH_3$				−515.936669	−515.9347409	−515.9345424	−516.3094498	−516.3075475	−516.3073888	0.069331593	0.070911576	0.070789341
I				−660.3549228	−660.3536746	−660.3534176	−660.7352195	−660.7341597	−660.7338433	0.067956029	0.069061742	0.068074416
NH_2				−418.6642568	−418.6630132	−418.6627332	−419.0689948	−419.0682696	−419.0680229	0.056266151	0.057542082	0.057293271
H				−363.3070807	−363.3061928	−363.3060198	−363.6869131	−363.6862611	−363.6859749	0.06709095	0.068298106	0.068021219
SO_3^-				−986.7039639	−986.7012746	−986.7012617	−987.188472	−987.1856892	−987.1858648	−0.04283444	−0.039429498	−0.038350523
				C0	C1	C2	C0	C1	C2	P4	P5	P6
C_4H_9	M062X/ def2QZVP	1	NH -HO	−520.4619515	−520.4596445	−520.4595946	−520.8462525	−520.844276	−520.8441198	0.060173819	0.061242085	0.060949428
CF_3				−700.4295201	−700.4244444	−700.4256213	−700.7902076	−700.7871476	−700.7875364	0.072314686	0.074814912	0.074066267
CH_3O				−477.8229707	−477.8213297	−477.8212917	−478.2124824	−478.2114273	−478.2111799	0.058188639	0.059680957	0.058993323
CH_3				−402.6054307	−402.6027596	−402.6030049	−402.9864482	−402.9852032	−402.9848921	0.06254247	0.063835063	0.063275393
CN				−455.5706977	−455.5689004	−455.5689284	−455.9302225	−455.92935	−455.9289943	0.074703873	0.077101916	0.076307378
$COCH_3$				−515.9406667	−515.9382532	−515.9382645	−516.3094498	−516.3075475	−516.3073888	0.068026151	0.069951473	0.069479622
I				−660.3590502	−660.3574376	−660.3573433	−660.7352195	−660.7341597	−660.7338433	0.066043771	0.067653937	0.066444445
NH_2				−418.6719505	−418.6702999	−418.6702006	−419.0689948	−419.0682696	−419.0680229	0.055821807	0.057343763	0.057028351
H				−363.3121045	−363.310536	−363.3106209	−363.6869131	−363.6862611	−363.6859749	0.06596643	0.067547164	0.066843733

SO_3^-				-986.7179746	-986.7150984	-986.7152947	-987.188472	-987.1856892	-987.1858648	-0.03203978	-0.029274256	-0.028925672
				C0	C1	C2	C0	C1	C2	P4	P5	P6
C_4H_9	M062X/ def2QZVP	2	NHHO -2HO	-596.8893116	-596.8848273	-596.8849157	-597.2851202	-597.282257	-597.2818281	0.04859489	0.050347679	0.050047837
CF_3				-776.8561131	-776.8510549	-776.8514226	-777.2310544	-777.2280367	-777.2278006	0.059989889	0.063133326	0.062485864
CH_3O				-554.2504118	-554.2467741	-554.246786	-554.6513842	-554.6492087	-554.6487187	0.04673897	0.048906616	0.048316184
CH_3				-479.0335561	-479.0285677	-479.0289588	-479.4270682	-479.424015	-479.4237905	0.050499786	0.052499207	0.05196183
CN				-531.9991799	-531.9959797	-531.995823	-532.3712162	-532.369669	-532.3690869	0.062130976	0.06519473	0.064379289
$COCH_3$				-592.3693464	-592.3644126	-592.3646795	-592.7509646	-592.7474627	-592.7473763	0.055875124	0.058483085	0.057970764
I				-736.7868492	-736.7834871	-736.7833922	-737.1742045	-737.1721799	-737.1716769	0.054701741	0.056848043	0.055736672
NH_2				-495.0982401	-495.0949425	-495.0948043	-495.5072151	-495.5048363	-495.5044918	0.044474966	0.046706693	0.046410862
H				-439.7389793	-439.736463	-439.7362404	-440.1269024	-440.1253786	-440.1248515	0.053441546	0.055742327	0.0551133
SO_3^-				-1063.140129	-1063.135571	-1063.135633	-1063.620219	-1063.617108	-1063.616815	-0.04139573	-0.03824636	-0.03751406
				C0	C1	C2	C0	C1	C2	P4	P5	P6
C_4H_9	M062X/ def2QZVP	2	NHA90 -HOA90	-596.8890361	-596.885041	-596.8850267	-597.2691002	-597.2652689	-597.2652335	0.062496704	0.063029795	0.062870989
CF_3				-776.8517118	-776.8471912	-776.8473609	-777.2234487	-777.2181776	-777.218622	0.062152462	0.064823151	0.06345312
CH_3O				-554.2504574	-554.2467716	-554.2466868	-554.6387833	-554.6360585	-554.6356682	0.055829718	0.057109745	0.056271244
CH_3				-479.0316652	-479.0277863	-479.0276733	-479.4134729	-479.41064	-479.4102643	0.058995626	0.060260763	0.059501411
CN				-531.9957178	-531.9917987	-531.9918616	-532.3626024	-532.3602288	-532.3599712	0.063759827	0.06643463	0.064867882
$COCH_3$				-592.3666484	-592.3623038	-592.3622618	-592.7401073	-592.736903	-592.7368005	0.058156383	0.060542768	0.059253967
I				-736.7855974	-736.7815654	-736.7817389	-737.1650566	-737.1620545	-737.1617691	0.058881479	0.060531146	0.05900577
NH_2				-495.0987545	-495.0954948	-495.0953555	-495.4921976	-495.4897568	-495.4893301	0.058996698	0.06000943	0.060044095
H				-439.7394802	-439.7357673	-439.7358211	-440.1160829	-440.1138518	-440.1134796	0.060184932	0.061781828	0.060594901
SO_3^-				-1063.154664	-1063.150557	-1063.15075	-1063.621654	-1063.617311	-1063.617747	-0.02844569	-0.02655833	-0.02591759
				C0	C1	C2	C0	C1	C2	P4	P5	P6
C_4H_9	M062X/ def2QZVP	2	HOA90 -HOA90	-596.8842675	-596.8810988	-596.8807029	-597.2691002	-597.2652689	-597.2652335	0.064614485	0.064448145	0.064668743
CF_3				-776.8513389	-776.846205	-776.8464877	-777.2234487	-777.2181776	-777.218622	0.065327228	0.067310681	0.066344484

CH_3O				-554.2447561	-554.2420977	-554.2416478	-554.6387833	-554.6360585	-554.6356682	0.058265915	0.058825694	0.058464436
CH_3				-479.0276552	-479.0243667	-479.0239606	-479.4134729	-479.41064	-479.4102643	0.061807512	0.06228382	0.061998168
CN				-531.9929163	-531.9903773	-531.9899461	-532.3626024	-532.3602288	-532.3599712	0.066346471	0.068384122	0.067090568
$COCH_3$				-592.3633835	-592.3596606	-592.3593564	-592.7401073	-592.736903	-592.7368005	0.060614079	0.062297618	0.061439815
I				-736.7813337	-736.7785264	-736.7781863	-737.1650566	-737.1620545	-737.1617691	0.061734674	0.062706713	0.061480933
NH_2				-495.0910235	-495.0889808	-495.0886102	-495.4921976	-495.4897568	-495.4893301	0.061091002	0.061522538	0.061886197
H				-439.7351922	-439.7324485	-439.7322417	-440.1160829	-440.1138518	-440.1134796	0.062773318	0.063703767	0.063022327
SO_3^-				-1063.142043	-1063.13835	-1063.138342	-1063.621654	-1063.617311	-1063.617747	-0.0371892	-0.034959	-0.03363663
				C0	C1	C2	C0	C1	C2	P4	P5	P6
C_4H_9	M062X/ def2QZVP	3	NHHOA90 -2HOA90	-673.3154286	-673.3104723	-673.3102698	-673.7087527	-673.7050963	-673.704487	0.050382532	0.051775865	0.051514041
CF_3				-853.2804739	-853.2742728	-853.274415	-853.6624721	-853.6568528	-853.656883	0.052474794	0.055388657	0.054187354
CH_3O				-630.6776445	-630.6721723	-630.6721683	-631.0769284	-631.072526	-631.0722129	0.046845956	0.048569136	0.04796693
CH_3				-555.457794	-555.4529076	-555.4527786	-555.8520264	-555.8481654	-555.8475976	0.05001214	0.051714609	0.051161702
CN				-608.4239764	-608.4192723	-608.419032	-608.8020311	-608.7982338	-608.7979496	0.054953977	0.057928704	0.056665566
$COCH_3$				-668.7950759	-668.7891343	-668.789121	-669.179127	-669.174362	-669.1740933	0.05111474	0.053507319	0.052647553
I				-813.2133401	-813.2076858	-813.2078452	-813.6025999	-813.5989139	-813.5983485	0.050020005	0.052025389	0.050682324
NH_2				-571.5248501	-571.5205117	-571.5202458	-571.9299089	-571.9246669	-571.9248471	0.049411459	0.051166945	0.051173944
H				-516.1659182	-516.1615747	-516.1613942	-516.5548452	-516.551843	-516.5513619	0.050628719	0.052596427	0.051651617
SO_3^-				-1139.576894	-1139.570932	-1139.5713	-1140.053463	-1140.048962	-1140.048863	-0.03765053	-0.03524752	-0.0344102
				C0	C1	C2	C0	C1	C2	P4	P5	P6
C_4H_9	M062X/ def2QZVP	4	NHHO2A90 -2HO2A90	-749.743974	-749.7359779	-749.7361662	-750.1331335	-750.1276716	-750.1270701	0.051319829	0.052529198	0.05222643
CF_3				-929.7035767	-929.6964	-929.6963084	-930.0907104	-930.0837502	-930.0836296	0.046270744	0.049121059	0.04739973
CH_3O				-707.1017344	-707.095545	-707.095565	-707.5039976	-707.499158	-707.4987291	0.041251608	0.043104617	0.04209295
CH_3				-631.8857346	-631.8782288	-631.8782465	-632.2779937	-632.2714395	-632.2710301	0.050869958	0.052298663	0.051839544
CN				-684.8467357	-684.8408599	-684.8403721	-685.2332798	-685.227471	-685.226975	0.046772794	0.049857631	0.047883533
$COCH_3$				-745.2209689	-745.2125858	-745.2126904	-745.6074226	-745.6004789	-745.6002495	0.046800588	0.049105468	0.047948074

I				-889.6402344	-889.6323092	-889.6323072	-890.0302391	-890.0237182	-890.0236029	0.046901001	0.048755154	0.047394144
NH_2				-647.9511861	-647.9452544	-647.9451304	-648.3529093	-648.3472792	-648.3467479	0.053030712	0.054156573	0.054642338
H				-592.592185	-592.5856319	-592.5855778	-592.9806351	-592.9756883	-592.9749404	0.049212816	0.050954375	0.050028234
SO_3^-				-1216.007855	-1216.000472	-1216.000631	-1216.48203	-1216.475572	-1216.475559	-0.03675938	-0.03479603	-0.03433858

Table S3. The calculated energies of R-PhNH$_2$ (A^-) and R-PhNH$_3^+$ (AH) based on direct method (unit: a.u.).

R	Calculation method	Number of H_2O	Models of A^--HA	A^-			AH		
				C0	C1	C2	C0	C1	C2
C_4H_9	CBS-QB3	0	/	-443.9951412	-443.9946573	-443.9944949	-444.4313436	-444.4307842	-444.4305900
CF_3				-623.8422811	-623.839322	-623.8402711	-624.2718605	-624.2685508	-624.2696913
CH_3O				-401.4409216	-401.4407477	-401.4406401	-401.8784559	-401.8780040	-401.8778018
CH_3				-326.3114415	-326.3099985	-326.310339	-326.7468160	-326.7462346	-326.7461387
CN				-379.2114288	-379.2114288	-379.2112987	-379.6392179	-379.6392179	-379.6390163
$COCH_3$				-439.5122866	-439.5118672	-439.5116847	-439.9417204	-439.9408158	-439.9407246
NH_2				-342.3622011	-342.3622011	-342.3621943	-342.8005325	-342.8005325	-342.8004488
H				-287.0784517	-287.0784517	-287.0784591	-287.5135837	-287.5135837	-287.5135217
SO_3^-				-909.8827743	-909.8809804	-909.881338	-910.3146758	-910.3132967	-910.3134647
				C0	C1	C2	C0	C1	C2
C_4H_9	CBS-QB3	1	HO-HO	-520.3510157	-520.3490415	-520.3488852	-520.7903847	-520.7883438	-520.7882381
CF_3				-700.1960480	-700.1934480	-700.1935307	-700.6279408	-700.6255284	-700.6256801
CH_3O				-477.7975009	-477.7955767	-477.7954599	-478.2369057	-478.2348550	-478.2349060
CH_3				-402.6666409	-402.6644996	-402.6644439	-403.1045648	-403.1031096	-403.1028910
CN				-455.5671852	-455.5658998	-455.5656309	-455.9963258	-455.9956287	-455.9953663
$COCH_3$				-515.8678319	-515.8661206	-515.8658987	-516.2991330	-516.2974791	-516.2973120
NH_2				-418.7181729	-418.7165132	-418.7164888	-419.1583489	-419.1573599	-419.1571894
H				-363.4343472	-363.4330580	-363.4329489	-363.8713345	-363.8703558	-363.8702285

SO_3^-				-986.2380381	-986.2355126	-986.2354881	-986.6738822	-986.6705181	-986.6708768
				C0	C1	C2	C0	C1	C2
C_4H_9				-596.7095327	-596.7044906	-596.7045933	-597.1507500	-597.1463157	-597.1466039
CF_3				-776.5547494	-776.549132	-776.5495094	-776.9903747	-776.9855202	-776.9857626
CH_3O				-554.153906	-554.1500992	-554.1499572	-554.5974475	-554.5931119	-554.5934164
CH_3				-479.0242333	-479.019376	-479.0195549	-479.4665678	-479.4618371	-479.4621438
CN	CBS-QB3	2	2HO-HOA90	-531.9235514	-531.9203364	-531.9203195	-532.3578027	-532.3549696	-532.3547883
$COCH_3$				-592.2268194	-592.2223977	-592.2224122	-592.6612434	-592.6570472	-592.6572027
NH_2				-495.0750837	-495.0715237	-495.0714254	-495.5202822	-495.5160896	-495.5163805
H				-439.7902014	-439.7872638	-439.7870892	-440.2322398	-440.2290641	-440.2291335
SO_3^-				-1062.594286	-1062.590083	-1062.590125	-1063.0343875	-1063.0293833	-1063.0298760
				C0	C1	C2	C0	C1	C2
C_4H_9				-596.7095327	-596.7044906	-596.7045933	-597.1493092	-597.1454128	-597.1454364
CF_3				-776.5547494	-776.549132	-776.5495094	-776.988857	-776.9838845	-776.9844044
CH_3O				-554.153906	-554.1500992	-554.1499572	-554.5948665	-554.591873	-554.5916982
CH_3				-479.0242333	-479.019376	-479.0195549	-479.4637001	-479.4604969	-479.4603381
CN	CBS-QB3	2	2HO-2HO	-531.9235514	-531.9203364	-531.9203195	-532.3562686	-532.3536843	-532.3535961
$COCH_3$				-592.2268194	-592.2223977	-592.2224122	-592.6601526	-592.6564743	-592.656423
NH_2				-495.0750837	-495.0715237	-495.0714254	-495.5176027	-495.5150449	-495.5148385
H				-439.7902014	-439.7872638	-439.7870892	-440.2307822	-440.227873	-440.227896
SO_3^-				-1062.594286	-1062.590083	-1062.590125	-1063.032298	-1063.028199	-1063.028307
				C0	C1	C2	C0	C1	C2
C_4H_9				-444.6806272	-444.6801432	-444.6799809	-445.1131452	-445.1125858	-445.1123916
CF_3	M062X/def2QZVP	0	/	-624.6558941	-624.652935	-624.6538841	-625.0805492	-625.0772395	-625.0783801
CH_3O				-402.040914	-402.04074	-402.0406325	-402.4757212	-402.4752693	-402.4750671
CH_3				-326.8253161	-326.8238731	-326.8242136	-327.2570586	-327.2564772	-327.2563813

CN				-379.8034899	-379.8034899	-379.8033598	-380.2255312	-380.2255312	-380.2253296
$COCH_3$				-440.1736086	-440.1731891	-440.1730066	-440.5977441	-440.5968395	-440.5967483
I				-584.5834192	-584.5834192	-584.5832879	-585.0122932	-585.0122809	-585.0120786
NH_2				-342.8880569	-342.8880569	-342.88805	-343.3252641	-343.3252641	-343.3251804
H				-287.5350289	-287.5350289	-287.5350363	-287.9658755	-287.9658755	-287.9658135
SO_3^-				-911.0344321	-911.0326381	-911.0329958	-911.4616509	-911.4602718	-911.4604398
				C0	C1	C2	C0	C1	C2
C_4H_9	M062X/def2QZVP	1	HO-HO	-521.1148007	-521.1128264	-521.1126702	-521.5514566	-521.5490428	-521.5490066
CF_3				-701.0880116	-701.0854116	-701.0854943	-701.5174767	-701.5147026	-701.5147585
CH_3O				-478.475974	-478.4740497	-478.473933	-478.913469	-478.9115364	-478.9114829
CH_3				-403.2591848	-403.2570436	-403.2569878	-403.6958849	-403.693167	-403.6933971
CN				-456.2378523	-456.2365668	-456.236298	-456.6652667	-456.6631975	-456.6631769
$COCH_3$				-516.6076706	-516.6059593	-516.6057374	-517.0378633	-517.0341671	-517.0346941
I				-661.0177494	-661.0162067	-661.0160018	-661.4504691	-661.4490078	-661.4488448
NH_2				-419.3235028	-419.3218431	-419.3218187	-419.7634013	-419.7618842	-419.7618696
H				-363.9697146	-363.9684255	-363.9683163	-364.4030434	-364.4020677	-364.4019407
SO_3^-				-987.468005	-987.4654795	-987.465455	-987.9008752	-987.8976502	-987.8978804
				C0	C1	C2	C0	C1	C2
C_4H_9	M062X/def2QZVP	1	NH-HO	-521.1187622	-521.1155705	-521.1159321	-521.5514566	-521.5490428	-521.5490066
CF_3				-701.0908014	-701.0870498	-701.0876064	-701.5174767	-701.5147026	-701.5147585
CH_3O				-478.4787023	-478.4762552	-478.4765284	-478.913469	-478.9115364	-478.9114829
CH_3				-403.2635942	-403.2598291	-403.2603984	-403.6958849	-403.693167	-403.6933971
CN				-456.2395546	-456.2374096	-456.2374588	-456.6652667	-456.6631975	-456.6631769
$COCH_3$				-516.6101967	-516.6071913	-516.6073362	-517.0378633	-517.0341671	-517.0346941
I				-661.0206024	-661.0182033	-661.0183373	-661.4504691	-661.4490078	-661.4488448
NH_2				-419.3277187	-419.3249309	-419.325491	-419.7634013	-419.7618842	-419.7618696

H				-363.9729012	-363.9707057	-363.9709024	-364.4030434	-364.4020677	-364.4019407
SO_3^-				-987.4716978	-987.4673676	-987.4681271	-987.9008752	-987.8976502	-987.8978804
				C0	C1	C2	C0	C1	C2
C_4H_9	M062X/def2QZVP	2	HONH-HOA90	-597.5532383	-597.5484867	-597.5487121	-597.9900069	-597.9855727	-597.9858608
CF_3				-777.5244968	-777.5196527	-777.5200486	-777.9567387	-777.9518841	-777.9521265
CH_3O				-554.9140382	-554.9096108	-554.9100059	-555.3523531	-555.3480175	-555.3483221
CH_3				-479.6974492	-479.6926679	-479.6929224	-480.1347436	-480.1300129	-480.1303196
CN				-532.6734077	-532.6699373	-532.6699724	-533.1021722	-533.0993391	-533.0991578
$COCH_3$				-593.0441719	-593.0399639	-593.0399998	-593.4748014	-593.4706052	-593.4707607
I				-737.4550552	-737.4510745	-737.4512902	-737.8866942	-737.8843933	-737.8840767
NH_2				-495.7617308	-495.757879	-495.7582155	-496.2029039	-496.1987113	-496.1990022
H				-440.408057	-440.4039944	-440.4044377	-440.8427561	-440.8395804	-440.8396498
SO_3^-				-1063.904866	-1063.899977	-1063.90026	-1064.339065	-1064.334061	-1064.334553
				C0	C1	C2	C0	C1	C2
C_4H_9	M062X/def2QZVP	2	HONH-2HO	-597.5532383	-597.5484867	-597.5487121	-597.9886823	-597.9847859	-597.9848094
CF_3				-777.5244968	-777.5196527	-777.5200486	-777.9554807	-777.9505083	-777.9510282
CH_3O				-554.9140382	-554.9096108	-554.9100059	-555.3499769	-555.3469834	-555.3468086
CH_3				-479.6974492	-479.6926679	-479.6929224	-480.1320357	-480.1288325	-480.1286736
CN				-532.6734077	-532.6699373	-532.6699724	-533.1008015	-533.0982172	-533.0981291
$COCH_3$				-593.0441719	-593.0399639	-593.0399998	-593.4739839	-593.4703056	-593.4702543
I				-737.4550552	-737.4510745	-737.4512902	-737.8877645	-737.8847854	-737.884582
NH_2				-495.7617308	-495.757879	-495.7582155	-496.2002281	-496.1976702	-496.1974638
H				-440.408057	-440.4039944	-440.4044377	-440.8414458	-440.8385366	-440.8385596
SO_3^-				-1063.904866	-1063.899977	-1063.90026	-1064.337084	-1064.332985	-1064.333092
				C0	C1	C2	C0	C1	C2
C_4H_9	M062X/def2QZVP	2	2HO-HOA90	-597.5514913	-597.5464492	-597.5465518	-597.9900069	-597.9855727	-597.9858608

CF_3				-777.5249903	-777.5193729	-777.5197504	-777.9567387	-777.9518841	-777.9521265
CH_3O				-554.910707	-554.9069002	-554.9067583	-555.3523531	-555.3480175	-555.3483221
CH_3				-479.6951534	-479.6902962	-479.690475	-480.1347436	-480.1300129	-480.1303196
CN				-532.6727761	-532.6695611	-532.6695441	-533.1021722	-533.0993391	-533.0991578
$COCH_3$				-593.0450108	-593.0405891	-593.0406036	-593.4748014	-593.4706052	-593.4707607
I				-737.4525729	-737.4490853	-737.4489846	-737.8866942	-737.8843933	-737.8840767
NH_2				-495.7587964	-495.7552365	-495.7551382	-496.2029039	-496.1987113	-496.1990022
H				-440.404097	-440.4011593	-440.4009848	-440.8427561	-440.8395804	-440.8396498
SO_3^-				-1063.902529	-1063.898327	-1063.898369	-1064.339065	-1064.334061	-1064.334553
				C0	C1	C2	C0	C1	C2
C_4H_9				-597.5514913	-597.5464492	-597.5465518	-597.9886823	-597.9847859	-597.9848094
CF_3				-777.5249903	-777.5193729	-777.5197504	-777.9554807	-777.9505083	-777.9510282
CH_3O				-554.910707	-554.9069002	-554.9067583	-555.3499769	-555.3469834	-555.3468086
CH_3				-479.6951534	-479.6902962	-479.690475	-480.1320357	-480.1288325	-480.1286736
CN	M062X/def2QZVP	2	2HO-2HO	-532.6727761	-532.6695611	-532.6695441	-533.1008015	-533.0982172	-533.0981291
$COCH_3$				-593.0450108	-593.0405891	-593.0406036	-593.4739839	-593.4703056	-593.4702543
I				-737.4525729	-737.4490853	-737.4489846	-737.8877645	-737.8847854	-737.884582
NH_2				-495.7587964	-495.7552365	-495.7551382	-496.2002281	-496.1976702	-496.1974638
H				-440.404097	-440.4011593	-440.4009848	-440.8414458	-440.8385366	-440.8385596
SO_3^-				-1063.902529	-1063.898327	-1063.898369	-1064.337084	-1064.332985	-1064.333092
				C0	C1	C2	C0	C1	C2
C_4H_9				-673.9900095	-673.9817574	-673.9825356	-674.4262802	-674.420554	-674.4206041
CF_3				-853.9611128	-853.9528604	-853.9537904	-854.3933241	-854.3870171	-854.3872403
CH_3O	M062X/def2QZVP	2	2HONH-3HO	-631.3509785	-631.3434528	-631.3441383	-631.7875622	-631.7827594	-631.782808
CH_3				-556.1327443	-556.1258688	-556.1263256	-556.5698921	-556.5645491	-556.5646634
CN				-609.1096709	-609.103205	-609.1036292	-609.5392774	-609.5345274	-609.5345975

$COCH_3$				-669.4811985	-669.4731805	-669.4741076	-669.9126248	-669.9064511	-669.9066767
I				-813.890694	-813.8841989	-813.8846087	-814.3269321	-814.3203921	-814.3212501
NH_2				-572.1973926	-572.1912157	-572.1915959	-572.6392654	-572.6333449	-572.6337714
H				-516.8442679	-516.8379315	-516.8382983	-517.2793089	-517.27484	-517.2747599
SO_3^-				-1140.340337	-1140.333028	-1140.333526	-1140.77446	-1140.768859	-1140.768902

Table S4. The calculated energies of R-$PhNH_2$ (A^-) and R-$PhNH_3^+$ (AH) based on indirect method (unit: a.u.).

R	Calculation method	Number of H_2O	Models of A^--HA	A^-			AH			Solvation energy		
				C0	C1	C2	C0	C1	C2	P1	P2	P3
C_4H_9	CBS-QB3	0	/	-443.9890879	-443.9886040	-443.9884416	-444.3278091	-444.3272497	-444.3270555	0.095647	0.097344	0.097344
CF_3				-623.8351266	-623.8321675	-623.8331166	-624.1548140	-624.1515043	-624.1526448	0.107685	0.111332	0.111332
CH_3O				-401.4312930	-401.4311191	-401.4310115	-401.7715807	-401.7711288	-401.7709266	0.095287	0.097572	0.097572
CH_3				-326.3043709	-326.3029279	-326.3032684	-326.6407358	-326.6401544	-326.6400585	0.097143	0.098648	0.098648
CN				-379.1991545	-379.1991545	-379.1990244	-379.5143750	-379.5143750	-379.5141734	0.110762	0.113468	0.113468
$COCH_3$				-439.4982543	-439.4978349	-439.4976524	-439.8238970	-439.8229924	-439.8229012	0.102159	0.105404	0.105404
NH_2				-342.3481360	-342.3481360	-342.3481292	-342.6911755	-342.6911755	-342.6910918	0.093006	0.095188	0.095188
H				-287.0706295	-287.0706295	-287.0706369	-287.4042858	-287.4042858	-287.4042238	0.099581	0.101193	0.101193
SO_3^-				-909.7669577	-909.7651638	-909.7655214	-910.1947193	-910.1933402	-910.1935082	0.001174	-0.00079	0.005629
				C0	C1	C2	C0	C1	C2	P4	P5	P6
C_4H_9	CBS-QB3	1	HO<br>-HO	-520.3375482	-520.3355740	-520.3354177	-520.6951084	-520.6930675	-520.6929618	0.080929	0.079658	0.081851
CF_3				-700.1828427	-700.1802427	-700.1803254	-700.5211479	-700.5187355	-700.5188872	0.093929	0.091462	0.094802
CH_3O				-477.7801778	-477.7782536	-477.7781368	-478.1385193	-478.1364686	-478.1365196	0.080002	0.078512	0.080351
CH_3				-402.6519186	-402.6497773	-402.6497216	-403.0072431	-403.0057879	-403.0055693	0.080889	0.07956	0.081027
CN				-455.5491116	-455.5478262	-455.5475573	-455.8820302	-455.8813331	-455.8810707	0.096301	0.09405	0.097037
$COCH_3$				-515.8472211	-515.8455098	-515.8452879	-516.1905887	-516.1889348	-516.1887677	0.086432	0.084553	0.087287

NH_2				-418.6961313	-418.6944716	-418.6944472	-419.0571909	-419.0562019	-419.0560314	0.077851	0.07619	0.078131
H				-363.4190497	-363.4177605	-363.4176514	-363.7712622	-363.7702835	-363.7701562	0.083111	0.081547	0.083303
SO_3^-				-986.1100435	-986.107518	-986.1074935	-986.5561934	-986.5528293	-986.5531880	-0.01424	-0.01662	-0.01142
				C0	C1	C2	C0	C1	C2	P4	P5	P6
C_4H_9				-596.6872116	-596.6821695	-596.6822722	-597.0597942	-597.0553599	-597.0556481	0.065507	0.063013	0.066132
CF_3				-776.5341576	-776.5285402	-776.5289176	-776.8877217	-776.8828672	-776.8831096	0.079473	0.076012	0.080412
CH_3O				-554.1280300	-554.1242232	-554.1240812	-554.5036941	-554.4993585	-554.4996630	0.065087	0.062578	0.065457
CH_3				-479.0008566	-478.9959993	-478.9961782	-479.3738104	-479.3690797	-479.3693864	0.066366	0.063643	0.066847
CN	CBS-QB3	2	2HO<br>-HOA90	-531.8985210	-531.8953060	-531.8952891	-532.2490412	-532.2462081	-532.2460268	0.081152	0.077916	0.08217
$COCH_3$				-592.1983120	-592.1938903	-592.1939048	-592.5566340	-592.5524378	-592.5525933	0.073727	0.071185	0.074581
NH_2				-495.0445659	-495.0410059	-495.0409076	-495.4220032	-495.4178106	-495.4181015	0.064756	0.062158	0.065264
H				-439.7665839	-439.7636463	-439.7634717	-440.1372014	-440.1340257	-440.1340951	0.068305	0.065302	0.069145
SO_3^-				-1062.4546915	-1062.4504887	-1062.4505311	-1062.9189295	-1062.9139253	-1062.9144180	-0.02789	-0.03123	-0.02517
				C0	C1	C2	C0	C1	C2	P4	P5	P6
C_4H_9				-596.6872116	-596.6821695	-596.6822722	-597.0595086	-597.0556122	-597.0556358	0.064566	0.062182	0.064856
CF_3				-776.5341576	-776.5285402	-776.5289176	-776.8889719	-776.8839994	-776.8845193	0.077022	0.073609	0.077408
CH_3O				-554.1280300	-554.1242232	-554.1240812	-554.5020807	-554.4990872	-554.4989124	0.064318	0.061889	0.064373
CH_3				-479.0008566	-478.9959993	-478.9961782	-479.3720583	-479.3688551	-479.3686963	0.065425	0.062789	0.06557
CN	CBS-QB3	2	2HO<br>-2HO	-531.8985210	-531.8953060	-531.8952891	-532.2491837	-532.2465994	-532.2465112	0.07961	0.07628	0.080274
$COCH_3$				-592.1983120	-592.1938903	-592.1939048	-592.5574845	-592.5538062	-592.5537549	0.071922	0.069299	0.072403
NH_2				-495.0445659	-495.0410059	-495.0409076	-495.4211110	-495.4185532	-495.4183468	0.063001	0.060214	0.063185
H				-439.7665839	-439.7636463	-439.7634717	-440.1367710	-440.1338618	-440.1338848	0.06748	0.064519	0.068002
SO_3^-				-1062.4546915	-1062.4504887	-1062.4505311	-1062.9153775	-1062.9112783	-1062.9113860	-0.02635	-0.02945	-0.02385
				C0	C1	C2	C0	C1	C2	P1	P2	P3
C_4H_9	M062X<br>/def2QZVP	0	/	-444.674016	-444.6735321	-444.6733697	-445.0102049	-445.0096455	-445.0094513	0.095647	0.097344	0.097344
CF_3				-624.6482666	-624.6453075	-624.6462566	-624.9635593	-624.9602496	-624.9613901	0.107685	0.111332	0.111332

CH_3O				-402.0309541	-402.0307801	-402.0306726	-402.3694726	-402.3690206	-402.3688185	0.095287	0.097572	0.097572
CH_3				-326.8177694	-326.8163264	-326.8166669	-327.151523	-327.1509416	-327.1508457	0.097143	0.098648	0.098648
CN				-379.7899065	-379.7899065	-379.7897765	-380.0999628	-380.0999628	-380.0997612	0.110762	0.113468	0.113468
$COCH_3$				-440.1578358	-440.1574163	-440.1572338	-440.4788954	-440.4779908	-440.4778996	0.102159	0.105404	0.105404
I				-584.5737833	-584.5737833	-584.5736521	-584.8984106	-584.8983983	-584.898196	0.104469	0.105428	0.105428
NH_2				-342.8737248	-342.8737248	-342.8737179	-343.2165832	-343.2165832	-343.2164996	0.093006	0.095188	0.095188
H				-287.5267416	-287.5267416	-287.526749	-287.8569036	-287.8569036	-287.8568415	0.099581	0.101193	0.101193
SO_3^-				-910.9166166	-910.9148227	-910.9151804	-911.3411568	-911.3397777	-911.3399457	0.001174	-0.00079	0.005629
				C0	C1	C2	C0	C1	C2	P4	P5	P6
C_4H_9				-521.1005763	-521.098602	-521.0984458	-521.4554276	-521.4530138	-521.4529776	0.080929	0.079658	0.081851
CF_3				-701.0742089	-701.0716089	-701.0716916	-701.4089941	-701.4062199	-701.4062759	0.093929	0.091462	0.094802
CH_3O				-478.4582153	-478.456291	-478.4561743	-478.8149667	-478.8130341	-478.8129806	0.080002	0.078512	0.080351
CH_3				-403.2438869	-403.2417457	-403.24169	-403.5990561	-403.5963382	-403.5965683	0.080889	0.07956	0.081027
CN	M062X /def2QZVP	1	HO -HO	-456.2183821	-456.2170967	-456.2168279	-456.5489223	-456.5468531	-456.5468326	0.096301	0.09405	0.097037
$COCH_3$				-516.5851886	-516.5834773	-516.5832554	-516.9282515	-516.9245553	-516.9250823	0.086432	0.084553	0.087287
I				-661.0015254	-660.9999827	-660.9997778	-661.3456887	-661.3442274	-661.3440644	0.088285	0.086665	0.088437
NH_2				-419.3010757	-419.299416	-419.2993916	-419.6622204	-419.6607034	-419.6606887	0.077851	0.07619	0.078131
H				-363.9538714	-363.9525823	-363.9524731	-364.303387	-364.3024112	-364.3022843	0.083111	0.081547	0.083303
SO_3^-				-987.3378525	-987.335327	-987.3353026	-987.7833217	-987.7800967	-987.7803269	-0.01424	-0.01662	-0.01142
				C0	C1	C2	C0	C1	C2	P4	P5	P6
C_4H_9				-521.1028444	-521.0996527	-521.1000143	-521.4554276	-521.4530138	-521.4529776	0.079042	0.0779	0.079822
CF_3				-701.0731251	-701.0693735	-701.0699301	-701.4089941	-701.4062199	-701.4062759	0.089817	0.087449	0.090168
CH_3O	M062X /def2QZVP	1	NH -HO	-478.4594967	-478.4570496	-478.4573228	-478.8149667	-478.8130341	-478.8129806	0.07814	0.076599	0.078653
CH_3				-403.2466827	-403.2429177	-403.2434869	-403.5990561	-403.5963382	-403.5965683	0.078862	0.077472	0.079154
CN				-456.2157373	-456.2135924	-456.2136416	-456.5489223	-456.5468531	-456.5468326	0.091655	0.089362	0.091759
$COCH_3$				-516.5848981	-516.5818927	-516.5820377	-516.9282515	-516.9245553	-516.9250823	0.083424	0.081543	0.083666

I				-660.9998885	-660.9974894	-660.9976234	-661.3456887	-661.3442274	-661.3440644	0.083089	0.081404	0.082988
NH_2				-419.3041729	-419.301385	-419.3019452	-419.6622204	-419.6607034	-419.6606887	0.076347	0.07469	0.076871
H				-363.9552566	-363.953061	-363.9532577	-364.303387	-364.3024112	-364.3022843	0.080947	0.079325	0.081156
SO_3^-				-987.3508855	-987.3465553	-987.3473149	-987.7833217	-987.7800967	-987.7803269	-0.00443	-0.00586	-0.00214
				C0	C1	C2	C0	C1	C2	P4	P5	P6
C_4H_9	M062X /def2QZVP	2	HONH -HOA90	-597.5307746	-597.5260229	-597.5262484	-597.899676	-597.8952418	-597.89553	0.066209	0.064201	0.067048
CF_3				-777.5019731	-777.497129	-777.497525	-777.85461	-777.8497554	-777.8499978	0.078064	0.074911	0.078788
CH_3O				-554.8881956	-554.8837682	-554.8841633	-555.2592551	-555.2549195	-555.2552241	0.065662	0.063631	0.066157
CH_3				-479.6741403	-479.6693589	-479.6696135	-480.042554	-480.0378233	-480.03813	0.0672	0.065013	0.067913
CN				-532.6436263	-532.6401559	-532.640191	-532.9931001	-532.9902671	-532.9900858	0.077538	0.07446	0.077877
$COCH_3$				-593.0135076	-593.0092997	-593.0093355	-593.369619	-593.3654227	-593.3655782	0.07306	0.070411	0.073895
I				-737.4303545	-737.4263738	-737.4265895	-737.7874346	-737.7851338	-737.7848172	0.073459	0.071226	0.073727
NH_2				-495.7313363	-495.7274844	-495.7278209	-496.1053969	-496.1012043	-496.1014952	0.065425	0.063364	0.066071
H				-440.3838498	-440.3797872	-440.3802305	-440.7481761	-440.7450004	-440.7450698	0.068803	0.066618	0.069182
SO_3^-				-1063.773196	-1063.768307	-1063.76859	-1064.222743	-1064.217739	-1064.218231	-0.0172	-0.01957	-0.01477
				C0	C1	C2	C0	C1	C2	P4	P5	P6
C_4H_9	M062X /def2QZVP	2	HONH -2HO	-597.5307746	-597.5260229	-597.5262484	-597.8994383	-597.8955418	-597.8955654	0.065268	0.06337	0.065772
CF_3				-777.5019731	-777.497129	-777.497525	-777.8559123	-777.8509399	-777.8514598	0.075613	0.072508	0.075783
CH_3O				-554.8881956	-554.8837682	-554.8841633	-555.257798	-555.2548045	-555.2546297	0.064893	0.062941	0.065073
CH_3				-479.6741403	-479.6693589	-479.6696135	-480.0409268	-480.0377236	-480.0375647	0.06626	0.064159	0.066637
CN				-532.6436263	-532.6401559	-532.640191	-532.9933381	-532.9907539	-532.9906657	0.075996	0.072824	0.075981
$COCH_3$				-593.0135076	-593.0092997	-593.0093355	-593.37065	-593.3669718	-593.3669205	0.071254	0.068525	0.071717
I				-737.4303545	-737.4263738	-737.4265895	-737.7897864	-737.7868073	-737.7866039	0.072208	0.070069	0.072233
NH_2				-495.7313363	-495.7274844	-495.7278209	-496.1045136	-496.1019557	-496.1017493	0.063669	0.06142	0.063992
H				-440.3838498	-440.3797872	-440.3802305	-440.7478312	-440.744922	-440.744945	0.067979	0.065835	0.068039
SO_3^-				-1063.773196	-1063.768307	-1063.76859	-1064.219411	-1064.215312	-1064.21542	-0.01567	-0.0178	-0.01344

				C0	C1	C2	C0	C1	C2	P4	P5	P6
C_4H_9	M062X /def2QZVP	2	2HO -HOA90	-597.5282859	-597.5232438	-597.5233465	-597.899676	-597.8952418	-597.89553	0.065507	0.063013	0.066132
CF_3				-777.5037213	-777.4981039	-777.4984814	-777.85461	-777.8497554	-777.8499978	0.079473	0.076012	0.080412
CH_3O				-554.8842145	-554.8804077	-554.8802657	-555.2592551	-555.2549195	-555.2552241	0.065087	0.062578	0.065457
CH_3				-479.6709849	-479.6661277	-479.6663065	-480.042554	-480.0378233	-480.03813	0.066366	0.063643	0.066847
CN				-532.6462102	-532.6429952	-532.6429782	-532.9931001	-532.9902671	-532.9900858	0.081152	0.077916	0.08217
$COCH_3$				-593.0146886	-593.0102668	-593.0102813	-593.369619	-593.3654227	-593.3655782	0.073727	0.071185	0.074581
I				-737.4285229	-737.4250353	-737.4249347	-737.7874346	-737.7851338	-737.7848172	0.074211	0.071595	0.074628
NH_2				-495.7276946	-495.7241346	-495.7240363	-496.1053969	-496.1012043	-496.1014952	0.064756	0.062158	0.065264
H				-440.3796619	-440.3767243	-440.3765497	-440.7481761	-440.7450004	-440.7450698	0.068305	0.065302	0.069145
SO_3^-				-1063.760648	-1063.756445	-1063.756488	-1064.222743	-1064.217739	-1064.218231	-0.02789	-0.03123	-0.02517
				C0	C1	C2	C0	C1	C2	P4	P5	P6
C_4H_9	M062X /def2QZVP	2	2HO -2HO	-597.5282859	-597.5232438	-597.5233465	-597.8994383	-597.8955418	-597.8955654	0.064566	0.062182	0.064856
CF_3				-777.5037213	-777.4981039	-777.4984814	-777.8559123	-777.8509399	-777.8514598	0.077022	0.073609	0.077408
CH_3O				-554.8842145	-554.8804077	-554.8802657	-555.257798	-555.2548045	-555.2546297	0.064318	0.061889	0.064373
CH_3				-479.6709849	-479.6661277	-479.6663065	-480.0409268	-480.0377236	-480.0375647	0.065425	0.062789	0.06557
CN				-532.6462102	-532.6429952	-532.6429782	-532.9933381	-532.9907539	-532.9906657	0.07961	0.07628	0.080274
$COCH_3$				-593.0146886	-593.0102668	-593.0102813	-593.37065	-593.3669718	-593.3669205	0.071922	0.069299	0.072403
I				-737.4285229	-737.4250353	-737.4249347	-737.7897864	-737.7868073	-737.7866039	0.07296	0.070437	0.073134
NH_2				-495.7276946	-495.7241346	-495.7240363	-496.1045136	-496.1019557	-496.1017493	0.063001	0.060214	0.063185
H				-440.3796619	-440.3767243	-440.3765497	-440.7478312	-440.744922	-440.744945	0.06748	0.064519	0.068002
SO_3^-				-1063.760648	-1063.756445	-1063.756488	-1064.219411	-1064.215312	-1064.21542	-0.02635	-0.02945	-0.02385
				C0	C1	C2	C0	C1	C2	P4	P5	P6
C_4H_9	M062X /def2QZVP	2	2HONH -3HO	-673.9597089	-673.9514569	-673.9522351	-674.3398582	-674.334132	-674.334182	0.053983	0.051171	0.054604
CF_3				-853.9323658	-853.9241134	-853.9250433	-854.2965386	-854.2902316	-854.2904548	0.06599	0.061947	0.066705
CH_3O				-631.3172529	-631.3097272	-631.3104127	-631.6984066	-631.6936039	-631.6936524	0.053464	0.050823	0.053598

CH_3				-556.1014852	-556.0946097	-556.0950665	-556.4818464	-556.4765034	-556.4766177	0.054795	0.052085	0.05501
CN				-609.0733994	-609.0669336	-609.0673578	-609.4359017	-609.4311518	-609.4312218	0.06481	0.06093	0.065148
$COCH_3$				-669.4440582	-669.4360402	-669.4369673	-669.812454	-669.8062803	-669.8065059	0.061052	0.057581	0.061864
I				-813.8592842	-813.8527891	-813.8531989	-814.232425	-814.225885	-814.226743	0.06143	0.058562	0.061761
NH_2				-572.1593836	-572.1532067	-572.1535869	-572.5465361	-572.5406156	-572.5410421	0.052509	0.049704	0.052954
H				-516.8126136	-516.8062772	-516.806644	-517.1879987	-517.1835298	-517.1834497	0.057755	0.054956	0.057979
SO_3^-				-1140.196696	-1140.189388	-1140.189885	-1140.656856	-1140.651255	-1140.651298	-0.0285	-0.03165	-0.02604

Table S5. The experimental and calculated pK_a values of R-PhNH$_2^{•+}$.

R	Experimental pK_a	Calculated pK_a														
		Calculation method	Number of H_2O		Direct approach			Indirect approach								
					C0	C1	C2	C0			C1			C2		
								P1	P2	P3	P1	P2	P3	P1	P2	P3
C_4H_9	8.2	CBS-QB3	0		7.99	7.82	7.86	7.07	7.77	7.56	6.91	7.60	7.39	6.94	7.63	7.42
CF_3	4.8				4.44	4.22	4.31	3.15	4.72	2.87	2.93	4.50	2.65	3.02	4.59	2.74
CH_3O	9.6				9.37	9.38	9.38	8.77	9.62	9.06	8.78	9.63	9.06	8.78	9.64	9.07
CH_3	8.5				7.91	7.97	7.90	7.17	7.75	7.76	7.23	7.81	7.83	7.16	7.74	7.76
CN	4				4.10	3.95	3.98	2.88	3.97	2.52	2.73	3.82	2.36	2.76	3.85	2.39
$COCH_3$	6.1				5.01	4.75	4.85	4.15	5.49	3.59	3.88	5.22	3.33	3.98	5.32	3.43
NH_2	12				13.75	13.73	13.70	12.80	13.58	14.60	12.78	13.56	14.58	12.74	13.52	14.54
H	7.05				6.80	6.80	6.79	5.87	6.46	6.27	5.87	6.46	6.27	5.86	6.45	6.26
SO_3^-	5.8				6.25	6.20	6.20	3.63	10.18	10.18	3.58	10.13	10.13	3.59	10.13	10.13
RMSE					0.75	0.81	0.78	1.43	1.92	2.11	1.53	1.95	2.17	1.50	1.94	2.41
R	Experimental pK_a	Calculation method	Number of H_2O	Models of A^--HA	Direct approach			C0			C1			C2		
					C0	C1	C2	P4	P5	P6	P4	P5	P6	P4	P5	P6
C_4H_9	8.2	CBS-QB3	1	*HO*-HO	8.02	8.65	8.45	6.56	6.85	6.86	7.20	7.48	7.50	7.00	7.29	7.30
CF_3	4.8				5.15	5.43	5.39	3.26	4.28	4.14	3.54	4.56	4.42	3.51	4.52	4.38

CH_3O	9.6				9.82	10.36	10.24	8.59	9.11	8.94	9.12	9.65	9.47	9.01	9.53	9.36
CH_3	8.5				8.73	8.93	8.92	7.30	7.70	7.59	7.50	7.90	7.79	7.48	7.89	7.78
CN	4				4.99	5.14	5.10	3.24	4.19	3.98	3.40	4.35	4.14	3.35	4.30	4.09
$COCH_3$	6.1				6.03	6.04	6.06	4.53	5.26	5.20	4.54	5.27	5.21	4.56	5.29	5.23
NH_2	12				13.93	14.17	14.19	12.21	12.80	12.69	12.45	13.04	12.92	12.47	13.05	12.94
H	7.05				7.92	8.03	7.97	6.39	6.94	6.81	6.49	7.05	6.92	6.44	7.00	6.87
SO_3^-	5.8				7.37	7.33	7.41	3.93	5.50	5.99	3.89	5.45	5.95	3.97	5.54	6.04
RMSE					0.95	1.09	1.08	1.27	0.70	0.74	1.09	0.57	0.56	1.12	0.60	0.61
C_4H_9	8.2				6.61	6.76	6.71	4.78	5.27	5.13	4.93	5.42	5.29	4.88	5.37	5.24
CF_3	4.8				2.77	3.69	3.33	0.53	1.68	1.34	1.46	2.61	2.26	1.10	2.25	1.90
CH_3O	9.6				8.10	8.37	8.27	6.46	7.15	6.83	6.73	7.42	7.10	6.63	7.32	7.00
CH_3	8.5				6.26	6.80	6.66	4.43	5.03	4.77	5.09	5.69	5.43	4.83	5.43	5.17
CN	4	CBS-QB3	1	*NH*-HO	3.26	3.68	3.51	1.06	2.16	1.80	1.49	2.59	2.22	1.31	2.41	2.05
$COCH_3$	6.1				4.03	4.27	4.19	2.12	3.00	2.79	2.35	3.24	3.02	2.27	3.16	2.94
NH_2	12				11.01	11.44	11.37	8.86	9.56	9.42	9.29	9.99	9.84	9.22	9.92	9.78
H	7.05				5.45	5.87	5.70	3.69	4.41	4.09	4.11	4.84	4.51	3.94	4.66	4.34
SO_3^-	5.8				5.47	5.52	5.51	2.67	3.94	4.10	2.71	3.98	4.14	2.70	3.97	4.13
RMSE					1.57	1.19	1.32	3.34	2.56	2.78	2.96	2.18	2.39	3.10	2.32	2.53
C_4H_9	8.2				7.00	7.75	7.51	4.87	5.67	5.54	5.61	6.42	6.28	5.38	6.18	6.04
CF_3	4.8				3.96	4.90	4.62	1.35	2.79	2.49	2.28	3.73	3.43	2.01	3.45	3.15
CH_3O	9.6				8.57	9.24	9.01	6.55	7.55	7.28	7.23	8.22	7.95	6.99	7.99	7.72
CH_3	8.5				6.90	7.79	7.51	4.88	5.80	5.55	5.77	6.69	6.44	5.49	6.41	6.16
CN	4	CBS-QB3	2	*NHHO*-2HO	3.88	4.64	4.45	1.25	2.66	2.28	2.01	3.42	3.04	1.81	3.22	2.85
$COCH_3$	6.1				4.73	5.39	5.23	2.47	3.67	3.43	3.13	4.33	4.09	2.97	4.16	3.93
NH_2	12				12.00	12.42	12.33	9.56	10.58	10.45	9.98	11.01	10.87	9.89	10.91	10.78
H	7.05				6.54	7.00	6.86	4.27	5.33	5.04	4.73	5.79	5.50	4.59	5.65	5.36
SO_3^-	5.8				5.85	6.52	6.36	2.76	4.21	4.55	3.43	4.88	5.21	3.27	4.71	5.05
RMSE					0.94	0.52	0.60	2.98	1.93	2.11	2.32	1.28	1.47	2.51	1.47	1.65

R	Experimental pK_a	Calculation method	Number of H_2O		Direct approach			Indirect approach								
					C0	C1	C2	C0			C1			C2		
								P1	P2	P3	P1	P2	P3	P1	P2	P3
C_4H_9	8.2	M062X/def2 QZVP	0		8.60	8.44	8.47	8.68	9.38	9.17	8.51	9.21	9.00	8.55	9.24	9.04
CF_3	4.8				4.14	3.92	4.01	3.61	5.18	3.34	3.40	4.96	3.12	3.49	5.05	3.21
CH_3O	9.6				10.19	10.20	10.20	10.17	11.02	10.45	10.18	11.03	10.46	10.18	11.03	10.46
CH_3	8.5				8.61	8.67	8.60	8.62	9.20	9.21	8.68	9.26	9.28	8.61	9.19	9.21
CN	4				3.40	3.25	3.28	3.17	4.26	2.81	3.02	4.11	2.65	3.05	4.14	2.68
$COCH_3$	6.1				4.89	4.63	4.73	4.75	6.09	4.20	4.49	5.83	3.94	4.59	5.93	4.04
I	7.1				7.08	6.92	6.95	7.71	7.84	7.59	7.55	7.68	7.43	7.58	7.71	7.46
NH_2	12				13.86	13.84	13.80	13.79	14.56	15.59	13.77	14.55	15.57	13.73	14.51	15.53
H	7.05				6.71	6.71	6.70	6.52	7.11	6.92	6.52	7.11	6.92	6.52	7.11	6.91
SO_3^-	5.8				5.85	5.80	5.81	4.50	1.26	1.26	4.45	1.21	1.21	4.46	1.22	1.22
RMSE					0.84	0.91	0.87	1.00	1.78	2.07	1.07	1.78	2.13	1.03	1.77	2.10
R	Experimental pK_a	Calculation method	Number of H_2O	Models of A^-–HA	Direct approach			Indirect approach								
					C0	C1	C2	C0			C1			C2		
								P1	P2	P3	P1	P2	P3	P1	P2	P3
C_4H_9	8.2	M062X/def2 QZVP	1	*HO*–HO	8.70	9.34	9.14	8.17	8.46	8.48	8.81	9.10	9.11	8.61	8.90	8.91
CF_3	4.8				4.96	5.24	5.20	3.73	4.75	4.61	4.01	5.03	4.89	3.98	4.99	4.85
CH_3O	9.6				10.73	11.27	11.15	10.01	10.53	10.36	10.54	11.07	10.89	10.43	10.95	10.78
CH_3	8.5				9.46	9.67	9.65	8.81	9.21	9.10	9.01	9.42	9.31	9.00	9.40	9.29
CN	4				4.76	4.92	4.87	3.76	4.71	4.50	3.92	4.87	4.66	3.87	4.82	4.61
$COCH_3$	6.1				6.02	6.03	6.05	5.14	5.86	5.81	5.15	5.88	5.82	5.17	5.89	5.84
I	7.1				8.31	8.39	8.37	7.96	8.47	8.02	8.05	8.56	8.10	8.02	8.53	8.07
NH_2	12				14.67	14.91	14.92	13.83	14.41	14.30	14.06	14.65	14.54	14.08	14.67	14.55
H	7.05				8.21	8.32	8.27	7.35	7.90	7.78	7.46	8.01	7.89	7.41	7.96	7.83
SO_3^-	5.8				7.15	7.11	7.19	4.94	6.50	7.00	4.89	6.46	6.95	4.98	6.54	7.04
RMSE					1.28	1.49	1.46	0.85	1.05	0.97	0.96	1.23	1.15	0.93	1.20	1.12
C_4H_9	8.2	M062X/def2 QZVP	1	*NH*–HO	7.12	7.27	7.23	6.22	6.71	6.58	6.38	6.87	6.73	6.33	6.82	6.68
CF_3	4.8				2.49	3.42	3.05	0.95	2.10	1.75	1.87	3.02	2.68	1.51	2.66	2.32

CH_3O	9.6				8.79	9.06	8.96	7.71	8.39	8.08	7.98	8.66	8.35	7.88	8.57	8.25
CH_3	8.5				6.82	7.47	7.22	5.80	6.40	6.14	6.46	7.05	6.80	6.20	6.80	6.54
CN	4				2.89	3.32	3.14	1.51	2.61	2.25	1.94	3.04	2.67	1.76	2.86	2.50
$COCH_3$	6.1				3.93	4.16	4.08	2.70	3.58	3.37	2.93	3.82	3.60	2.86	3.74	3.52
I	7.1				6.02	6.28	6.17	5.18	5.92	5.37	5.44	6.18	5.62	5.34	6.08	5.52
NH_2	12				11.24	11.67	11.60	10.08	10.78	10.64	10.51	11.21	11.06	10.44	11.14	11.00
H	7.05				5.70	6.12	5.95	4.52	5.25	4.93	4.94	5.67	5.35	4.77	5.50	5.18
SO_3^-	5.8				5.30	5.34	5.34	3.46	4.73	4.89	3.50	4.77	4.93	3.49	4.76	4.92
RMSE					1.48	1.07	1.22	2.58	1.76	2.01	2.18	1.36	1.61	2.33	1.50	1.76
C_4H_9	8.2	M062X/def2 QZVP	2	*NHHO*-2HO	7.58	8.33	8.09	6.19	7.00	6.86	6.94	7.74	7.60	6.70	7.50	7.37
CF_3	4.8				3.86	4.80	4.53	1.83	3.28	2.98	2.77	4.22	3.92	2.50	3.94	3.64
CH_3O	9.6				9.29	9.96	9.73	7.71	8.71	8.44	8.38	9.38	9.11	8.15	9.15	8.88
CH_3	8.5				7.54	8.43	8.15	6.01	6.93	6.68	6.90	7.82	7.57	6.62	7.54	7.29
CN	4				3.35	4.11	3.91	1.48	2.89	2.52	2.24	3.65	3.28	2.05	3.46	3.08
$COCH_3$	6.1				4.73	5.39	5.22	3.01	4.21	3.98	3.67	4.87	4.64	3.51	4.71	4.47
I	7.1				6.39	7.00	6.82	5.11	6.10	5.59	5.73	6.71	6.20	5.54	6.53	6.02
NH_2	12				12.01	12.43	12.34	10.35	11.38	11.24	10.77	11.80	11.66	10.68	11.71	11.57
H	7.05				6.50	6.96	6.82	4.79	5.85	5.56	5.25	6.31	6.02	5.11	6.17	5.88
SO_3^-	5.8				5.80	6.47	6.31	3.57	5.01	5.35	4.23	5.68	6.02	4.07	5.52	5.85
RMSE					0.77	0.38	0.41	2.35	1.24	1.47	1.67	0.59	0.83	1.86	0.765	1.012
C_4H_9	8.2	M062X/def2 QZVP	2	*NHA90*-HOA90	5.89	5.96	5.95	5.34	5.59	5.52	5.42	5.66	5.59	5.41	5.65	5.58
CF_3	4.8				3.18	2.84	2.96	1.36	2.58	1.95	1.01	2.24	1.61	1.14	2.36	1.73
CH_3O	9.6				7.12	7.56	7.42	6.08	6.67	6.28	6.52	7.11	6.72	6.38	6.97	6.58
CH_3	8.5				5.60	6.08	5.96	4.54	5.12	4.77	5.02	5.60	5.25	4.90	5.48	5.13
CN	4				1.56	2.27	2.13	-0.14	1.09	0.37	0.57	1.80	1.08	0.43	1.66	0.94
$COCH_3$	6.1				1.94	2.46	2.43	0.31	1.41	0.81	0.83	1.93	1.34	0.81	1.90	1.31
I	7.1				4.46	4.93	4.72	3.40	4.16	3.46	3.88	4.63	3.93	3.67	4.42	3.72
NH_2	12				10.72	11.09	10.96	9.89	10.35	10.37	10.26	10.73	10.75	10.13	10.60	10.61
H	7.05				3.97	4.65	4.46	2.69	3.42	2.88	3.37	4.10	3.56	3.17	3.91	3.36
SO_3^-	5.8				4.83	4.72	4.84	3.50	4.36	4.66	3.39	4.26	4.55	3.50	4.37	4.66

RMSE					2.68	2.30	2.38	3.76	2.98	3.41	3.41	2.62	3.04	3.48	2.695	3.119
C_4H_9	8.2				8.52	8.21	8.38	8.51	8.43	8.54	8.21	8.13	8.23	8.37	8.29	8.40
CF_3	4.8				4.26	4.19	4.27	2.99	3.90	3.45	2.92	3.84	3.39	3.00	3.91	3.47
CH_3O	9.6				10.28	10.25	10.27	9.82	10.08	9.91	9.79	10.05	9.88	9.82	10.07	9.91
CH_3	8.5				8.15	8.36	8.37	7.67	7.89	7.76	7.88	8.10	7.97	7.90	8.12	7.98
CN	4	M062X/def2 QZVP	2	*HOA90*–HOA90	3.52	3.59	3.67	2.34	3.28	2.68	2.42	3.35	2.76	2.50	3.43	2.84
$COCH_3$	6.1				4.02	4.26	4.35	2.94	3.72	3.32	3.18	3.95	3.56	3.27	4.05	3.65
I	7.1				7.14	7.05	7.08	6.68	7.12	6.56	6.59	7.03	6.47	6.61	7.06	6.49
NH_2	12				14.73	14.55	14.53	14.41	14.60	14.77	14.22	14.42	14.59	14.20	14.40	14.56
H	7.05				6.63	6.87	6.79	5.85	6.28	5.97	6.09	6.51	6.20	6.01	6.44	6.12
SO_3^-	5.8				6.76	6.46	6.66	5.28	6.31	6.91	4.98	6.01	6.61	5.18	6.21	6.82
RMSE					1.25	1.12	1.11	1.57	1.24	1.50	1.48	1.12	1.37	1.43	1.095	1.354
C_4H_9	8.2				7.01	7.61	7.42	5.87	6.51	6.39	6.47	7.11	6.99	6.28	6.92	6.80
CF_3	4.8				3.67	3.94	3.89	1.62	2.96	2.41	1.89	3.23	2.68	1.84	3.18	2.63
CH_3O	9.6				8.34	8.83	8.69	6.98	7.78	7.50	7.48	8.27	7.99	7.33	8.13	7.85
CH_3	8.5				7.50	7.98	7.77	6.12	6.90	6.65	6.59	7.37	7.12	6.39	7.17	6.92
CN	4	M062X/def2 QZVP	3	*NHHOA90*–2HOA90	2.87	3.28	3.26	0.95	2.32	1.74	1.37	2.74	2.15	1.35	2.72	2.13
$COCH_3$	6.1				3.68	4.22	4.10	1.94	3.04	2.65	2.48	3.58	3.19	2.37	3.47	3.07
I	7.1				5.18	6.09	5.76	3.83	4.76	4.14	4.74	5.66	5.04	4.41	5.33	4.71
NH_2	12				12.11	11.69	11.90	10.82	11.63	11.63	10.41	11.21	11.22	10.61	11.42	11.42
H	7.05				5.53	6.15	6.01	3.96	4.87	4.43	4.58	5.48	5.05	4.44	5.34	4.91
SO_3^-	5.8				5.43	6.10	5.88	3.67	4.77	5.16	4.34	5.45	5.83	4.13	5.23	5.62
RMSE					1.44	0.94	1.07	2.84	1.89	2.24	2.37	1.41	1.77	2.49	1.53	1.89
C_4H_9	8.2				5.39	6.55	6.19	4.39	4.94	4.80	5.55	6.11	5.97	5.19	5.74	5.61
CF_3	4.8				3.21	3.31	3.29	1.13	2.44	1.65	1.23	2.54	1.75	1.22	2.53	1.74
CH_3O	9.6				7.22	7.84	7.64	5.78	6.63	6.17	6.40	7.26	6.79	6.20	7.05	6.58
CH_3	8.5	M062X/def2 QZVP	4	*NHHO2A90*–2HO2A90	6.84	7.27	7.08	5.60	6.26	6.05	6.04	6.70	6.49	5.85	6.50	6.29
CN	4				3.22	3.25	3.25	1.09	2.51	1.60	1.12	2.54	1.63	1.12	2.54	1.63
$COCH_3$	6.1				2.81	3.48	3.32	1.06	2.12	1.59	1.73	2.79	2.25	1.57	2.63	2.10
I	7.1				4.11	4.76	4.71	2.74	3.60	2.97	3.39	4.24	3.62	3.34	4.19	3.56

NH_2	12				11.90	12.04	11.85	10.95	11.47	11.69	11.09	11.61	11.83	10.90	11.42	11.64
H	7.05				4.57	5.31	4.99	3.09	3.89	3.47	3.83	4.63	4.20	3.51	4.31	3.89
SO_3^-	5.8				4.45	4.88	4.80	2.98	3.88	4.09	3.40	4.31	4.52	3.32	4.23	4.44
RMSE					2.29	1.71	1.87	3.58	2.72	3.13	3.07	2.19	2.62	3.22	2.338	2.761

Table S6. The experimental and calculated pK_a values of R-$PhNH_3^+$.

R	Experimental pK_a	Calculated pK_a														
		Calculation method	Number of H_2O		Direct approach			Indirect approach								
					C0	C1	C2	C0			C1			C2		
								P1	P2	P3	P1	P2	P3	P1	P2	**P3**
C_4H_9	4.95	CBS-QB3	0		2.42	2.38	2.37	1.58	2.36	2.36	1.54	2.32	2.32	1.53	2.31	**2.31**
CF_3	2.75				−0.63	−0.79	−0.70	−1.64	0.04	0.04	−1.80	−0.13	−0.13	−1.72	−0.04	**−0.04**
CH_3O	5.29				3.03	2.90	2.86	2.13	3.18	3.18	2.00	3.05	3.05	1.96	3.01	**3.01**
CH_3	5.12				2.04	2.43	2.23	1.18	1.87	1.87	1.58	2.27	2.27	1.38	2.07	**2.07**
CN	1.79				−1.45	−1.45	−1.48	−2.28	−1.04	−1.04	−2.28	−1.04	−1.04	−2.31	−1.07	**−1.07**
$COCH_3$	2.23				−0.69	−0.92	−0.88	−1.44	0.05	0.05	−1.67	−0.18	−0.18	−1.63	−0.13	**−0.13**
NH_2	6.07				3.40	3.40	3.36	2.35	3.35	3.35	2.35	3.35	3.35	2.31	3.32	**3.32**
H	4.60				1.93	1.93	1.89	1.06	1.80	1.80	1.06	1.80	1.80	1.02	1.76	**1.76**
SO_3^-	3.17				0.44	0.63	0.54	−0.92	−1.82	1.13	−0.73	−1.63	1.32	−0.82	−1.72	**1.23**
RMSE					2.85	2.85	2.88	3.79	3.02	2.61	3.79	2.99	2.60	3.82	3.03	**2.63**
R	Experimental pK_a	Calculation method	Number of H_2O	Models of A^--HA	Direct approach			C0			C1			C2		
					C0	C1	C2	P4	P5	P6	P4	P5	P6	P4	P5	**P6**
C_4H_9	4.95	CBS-QB3	1	*HO*-HO	3.88	3.84	3.87	3.47	2.89	3.89	3.44	2.86	3.86	3.46	2.88	**3.89**
CF_3	2.75				0.44	0.52	0.55	0.59	−0.54	1.00	0.68	−0.45	1.08	0.71	−0.42	**1.11**
CH_3O	5.29				3.89	3.83	3.91	3.40	2.72	3.56	3.35	2.66	3.51	3.42	2.74	**3.58**
CH_3	5.12				3.21	3.53	3.45	2.42	1.81	2.49	2.74	2.13	2.80	2.66	2.05	**2.73**
CN	1.79				−0.83	−0.56	−0.56	−0.79	−1.83	−0.45	−0.52	−1.56	−0.18	−0.52	−1.55	**−0.18**
$COCH_3$	2.23				0.16	0.19	0.22	−0.53	−1.39	−0.13	−0.50	−1.36	−0.11	−0.47	−1.34	**−0.08**
NH_2	6.07				4.25	4.55	4.49	3.66	2.90	3.79	3.97	3.21	4.10	3.91	3.14	**4.03**

H	4.60				2.78	2.92	2.91	2.01	1.30	2.10	2.16	1.44	2.25	2.15	1.43	**2.24**
SO_3^-	3.17				2.25	1.87	2.04	0.45	−0.65	1.74	0.06	−1.03	1.36	0.24	−0.86	**1.53**
RMSE					1.85	1.74	1.73	2.40	3.24	2.06	2.33	3.17	1.96	2.30	3.15	**1.95**
C_4H_9	4.95	CBS-QB3	2	*2HO*-HOA90	4.73	5.00	5.09	3.29	2.14	3.57	3.57	2.42	3.85	3.65	2.50	**3.94**
CF_3	2.75				2.15	2.50	2.44	0.96	−0.63	1.40	1.31	−0.28	1.75	1.25	−0.34	**1.68**
CH_3O	5.29				5.79	5.55	5.76	4.51	3.36	4.68	4.27	3.11	4.44	4.47	3.32	**4.64**
CH_3	5.12				5.24	5.30	5.36	3.85	2.60	4.07	3.91	2.66	4.13	3.97	2.72	**4.19**
CN	1.79				1.52	1.70	1.62	0.34	−1.15	0.80	0.51	−0.98	0.98	0.44	−1.05	**0.90**
$COCH_3$	2.23				1.60	1.70	1.77	0.51	−0.66	0.90	0.61	−0.56	1.01	0.68	−0.49	**1.07**
NH_2	6.07				6.56	6.27	6.44	5.17	3.98	5.41	4.88	3.69	5.12	5.06	3.87	**5.30**
H	4.60				5.10	4.99	5.11	3.67	2.29	4.06	3.56	2.18	3.95	3.67	2.29	**4.06**
SO_3^-	3.17				4.21	3.84	4.05	2.49	0.95	3.74	2.12	0.58	3.37	2.33	0.79	**3.57**
RMSE					0.55	0.35	0.45	1.31	2.60	1.00	1.26	2.58	0.91	1.19	2.51	**0.86**
C_4H_9	4.95	CBS-QB3	2	*2HO*-2HO	4.06	4.59	4.55	2.72	1.63	2.86	3.25	2.15	3.38	3.21	2.12	**3.35**
CF_3	2.75				1.46	1.75	1.82	0.41	−1.16	0.59	0.71	−0.86	0.88	0.77	−0.80	**0.95**
CH_3O	5.29				4.61	4.98	4.97	3.42	2.30	3.44	3.79	2.67	3.81	3.77	2.66	**3.80**
CH_3	5.12				3.92	4.68	4.53	2.61	1.40	2.68	3.38	2.16	3.44	3.22	2.01	**3.29**
CN	1.79				0.82	1.11	1.07	−0.31	−1.84	0.00	−0.02	−1.55	0.29	−0.05	−1.58	**0.25**
$COCH_3$	2.23				1.10	1.44	1.41	0.07	−1.14	0.29	0.41	−0.79	0.63	0.38	−0.82	**0.60**
NH_2	6.07				5.32	5.78	5.74	3.96	2.68	4.04	4.42	3.14	4.50	4.37	3.09	**4.45**
H	4.60				4.43	4.45	4.54	3.09	1.73	3.33	3.11	1.74	3.35	3.20	1.83	**3.44**
SO_3^-	3.17				3.25	3.30	3.33	1.56	0.13	2.71	1.61	0.18	2.76	1.64	0.21	**2.79**
RMSE					0.89	0.54	0.56	2.07	3.38	1.87	1.71	3.03	1.49	1.73	3.04	**1.51**
R	Experimental pK_a	Calculation method	Number of H_2O		Direct approach			Indirect approach								
								C0			C1			C2		
					C0	C1	C2	P1	P2	P3	P1	P2	P3	P1	P2	**P3**
C_4H_9	4.95	M062X/def2QZVP	0		0.72	0.69	0.68	0.41	1.19	1.19	0.38	1.16	1.16	0.36	1.14	**1.14**
CF_3	2.75				−2.89	−3.05	−2.96	−3.66	−1.99	−1.99	−3.82	−2.15	−2.15	−3.74	−2.06	**−2.06**
CH_3O	5.29				1.78	1.65	1.61	1.32	2.37	2.37	1.19	2.24	2.24	1.15	2.20	**2.20**
CH_3	5.12				0.37	0.76	0.56	−0.02	0.67	0.67	0.37	1.07	1.07	0.17	0.87	**0.87**

CN	1.79				−4.09	−4.09	−4.13	−4.66	−3.41	−3.41	−4.66	−3.41	−3.41	−4.69	−3.44	**−3.44**
$COCH_3$	2.23				−3.13	−3.35	−3.31	−3.55	−2.06	−2.06	−3.78	−2.28	−2.28	−3.73	−2.24	**−2.24**
I	3.78				−0.95	−0.96	−0.99	−0.85	−0.41	−0.41	−0.85	−0.41	−0.41	−0.89	−0.45	**−0.45**
NH_2	6.07				2.88	2.88	2.85	2.26	3.27	3.27	2.26	3.27	3.27	2.23	3.23	**3.23**
H	4.60				−0.04	−0.04	−0.08	−0.55	0.19	0.19	−0.55	0.19	0.19	−0.58	0.16	**0.16**
SO_3^-	3.17				−1.71	−1.52	−1.61	−2.40	−3.31	−0.36	−2.21	−3.11	−0.17	−2.30	−3.20	**−0.25**
RMSE					4.75	4.75	4.78	5.22	4.44	4.09	5.22	4.43	4.09	5.25	4.46	**4.12**
R	Experimental pK_a	Calculation method	Number of H_2O	Models of A^--HA	Direct approach			Indirect approach								
					C0	C1	C2	C0			C1			C2		
								P4	P5	P6	P4	P5	P6	P4	P5	**P6**
C_4H_9	4.95	M062X/def2QZVP	1	*HO*-HO	2.63	2.43	2.48	2.22	1.64	2.65	2.02	1.44	2.45	2.08	1.49	**2.50**
CF_3	2.75				−0.68	−0.76	−0.77	−1.02	−2.16	−0.62	−1.10	−2.24	−0.70	−1.12	−2.25	**−0.72**
CH_3O	5.29				3.01	3.01	3.04	2.67	1.99	2.83	2.67	1.98	2.83	2.70	2.01	**2.86**
CH_3	5.12				2.65	2.38	2.51	2.35	1.74	2.42	2.09	1.48	2.15	2.22	1.61	**2.28**
CN	1.79				−1.62	−1.98	−1.87	−1.89	−2.92	−1.55	−2.25	−3.28	−1.91	−2.13	−3.17	**−1.79**
$COCH_3$	2.23				−0.34	−1.26	−0.91	−0.67	−1.53	−0.27	−1.58	−2.44	−1.19	−1.23	−2.10	**−0.84**
I	3.78				0.82	0.85	0.87	0.69	−0.05	0.76	0.73	−0.02	0.80	0.75	0.00	**0.82**
NH_2	6.07				4.12	4.18	4.19	3.70	2.94	3.83	3.77	3.01	3.90	3.77	3.01	**3.90**
H	4.60				1.10	1.24	1.23	0.77	0.06	0.86	0.92	0.20	1.01	0.91	0.19	**1.00**
SO_3^-	3.17				0.89	0.56	0.68	0.13	−0.96	1.43	−0.19	−1.28	1.11	−0.07	−1.17	**1.23**
RMSE					2.77	2.97	2.88	3.12	3.95	2.80	3.31	4.15	2.99	3.23	4.06	**2.91**
C_4H_9	4.95	M062X/def2QZVP	1	*NH*-HO	0.81	1.16	0.98	0.31	−0.21	0.67	0.67	0.15	1.03	0.49	−0.04	**0.85**
CF_3	2.75				−1.96	−1.51	−1.74	−2.42	−3.51	−2.26	−1.97	−3.06	−1.81	−2.20	−3.29	**−2.04**
CH_3O	5.29				1.76	2.00	1.85	1.23	0.52	1.46	1.46	0.75	1.70	1.31	0.60	**1.55**
CH_3	5.12				0.62	1.10	0.95	0.13	−0.50	0.27	0.62	−0.02	0.75	0.46	−0.18	**0.59**
CN	1.79				−2.41	−2.37	−2.40	−2.81	−3.86	−2.76	−2.77	−3.83	−2.72	−2.80	−3.86	**−2.76**
$COCH_3$	2.23				−1.51	−1.82	−1.65	−1.92	−2.78	−1.80	−2.23	−3.10	−2.12	−2.06	−2.92	**−1.95**
I	3.78				−0.49	−0.06	−0.20	−0.94	−1.72	−0.99	−0.51	−1.29	−0.56	−0.65	−1.42	**−0.70**
NH_2	6.07				2.18	2.76	2.50	1.59	0.83	1.83	2.17	1.41	2.41	1.91	1.15	**2.15**
H	4.60				−0.37	0.19	0.04	−0.86	−1.60	−0.76	−0.30	−1.04	−0.20	−0.45	−1.19	**−0.35**

SO_3^-	3.17				-0.81	-0.30	-0.55	-1.35	-2.01	-0.30	-0.84	-1.50	0.21	-1.08	-1.74	**-0.03**
RMSE					4.21	3.88	4.01	4.70	5.48	4.47	4.36	5.15	4.14	4.49	5.28	**4.27**
C_4H_9	4.95	M062X/def2QZVP	2	*HONH*-HOA90	2.68	2.83	2.85	1.92	0.99	2.30	2.06	1.14	2.45	2.09	1.17	**2.48**
CF_3	2.75				0.60	0.59	0.52	-0.11	-1.56	0.22	-0.12	-1.57	0.22	-0.19	-1.64	**0.15**
CH_3O	5.29				3.39	3.43	3.39	2.66	1.72	2.89	2.70	1.77	2.93	2.66	1.72	**2.89**
CH_3	5.12				2.92	2.94	2.97	2.15	1.14	2.48	2.17	1.17	2.50	2.20	1.19	**2.52**
CN	1.79				-1.00	-0.71	-0.81	-1.81	-3.22	-1.65	-1.51	-2.93	-1.36	-1.61	-3.03	**-1.46**
$COCH_3$	2.23				-0.14	-0.14	-0.08	-0.81	-2.03	-0.43	-0.81	-2.03	-0.43	-0.75	-1.97	**-0.37**
I	3.78				0.32	1.09	0.85	-0.19	-1.21	-0.06	0.59	-0.44	0.71	0.34	-0.68	**0.47**
NH_2	6.07				4.71	4.55	4.53	3.93	2.98	4.23	3.77	2.82	4.07	3.75	2.80	**4.05**
H	4.60				1.73	2.14	1.96	1.01	0.00	1.18	1.41	0.41	1.59	1.24	0.24	**1.42**
SO_3^-	3.17				1.50	1.44	1.54	0.64	-0.45	1.76	0.59	-0.50	1.71	0.69	-0.40	**1.81**
RMSE					2.38	2.19	2.24	3.08	4.18	2.77	2.90	4.01	2.58	2.96	4.06	**2.64**
C_4H_9	4.95	M062X/def2QZVP	2	*HONH*-2HO	2.07	2.46	2.37	1.37	0.50	1.61	1.77	0.90	2.00	1.68	0.80	**1.91**
CF_3	2.75				0.02	-0.04	0.02	-0.64	-2.07	-0.56	-0.70	-2.13	-0.62	-0.64	-2.07	**-0.56**
CH_3O	5.29				2.30	2.96	2.70	1.63	0.74	1.72	2.29	1.40	2.38	2.03	1.13	**2.11**
CH_3	5.12				1.68	2.40	2.21	0.97	0.00	1.14	1.69	0.73	1.87	1.50	0.54	**1.68**
CN	1.79				-1.63	-1.22	-1.28	-2.41	-3.87	-2.41	-2.00	-3.46	-2.01	-2.06	-3.52	**-2.06**
$COCH_3$	2.23				-0.52	-0.28	-0.32	-1.17	-2.43	-0.96	-0.93	-2.18	-0.71	-0.97	-2.22	**-0.75**
I	3.78				0.81	1.27	1.08	0.32	-0.66	0.33	0.78	-0.20	0.79	0.59	-0.40	**0.60**
NH_2	6.07				3.47	4.07	3.82	2.72	1.68	2.86	3.31	2.28	3.46	3.06	2.03	**3.21**
H	4.60				1.12	1.66	1.46	0.47	-0.52	0.50	1.00	0.01	1.03	0.81	-0.18	**0.83**
SO_3^-	3.17				0.59	0.95	0.87	-0.18	-1.16	0.84	0.18	-0.80	1.21	0.10	-0.88	**1.13**
RMSE					3.00	2.57	2.70	3.68	4.77	3.51	3.25	4.34	3.08	3.38	4.47	**3.20**
C_4H_9	4.95	M062X/def2QZVP	2	*2HO*-HOA90	3.48	3.76	3.85	2.74	1.59	3.03	3.02	1.87	3.31	3.10	1.96	**3.39**
CF_3	2.75				0.37	0.72	0.66	-0.27	-1.86	0.16	0.08	-1.51	0.52	0.02	-1.57	**0.45**
CH_3O	5.29				4.92	4.68	4.88	4.22	3.07	4.39	3.98	2.83	4.15	4.19	3.03	**4.36**
CH_3	5.12				3.98	4.04	4.09	3.22	1.96	3.44	3.27	2.02	3.50	3.33	2.08	**3.55**
CN	1.79				-0.71	-0.54	-0.61	-1.33	-2.82	-0.87	-1.16	-2.65	-0.69	-1.23	-2.72	**-0.77**
$COCH_3$	2.23				-0.53	-0.43	-0.36	-1.05	-2.22	-0.66	-0.95	-2.12	-0.55	-0.88	-2.05	**-0.49**

I	3.78				1.46	2.01	1.91	1.00	−0.20	1.19	1.55	0.35	1.74	1.45	0.25	**1.64**
NH_2	6.07				6.05	5.76	5.94	5.30	4.10	5.53	5.01	3.81	5.24	5.18	3.99	**5.42**
H	4.60				3.55	3.44	3.55	2.70	1.32	3.09	2.59	1.21	2.98	2.71	1.32	**3.09**
SO_3^-	3.17				2.57	2.20	2.41	1.50	−0.04	2.75	1.13	−0.40	2.38	1.34	−0.20	**2.59**
RMSE					1.73	1.58	1.56	2.32	3.59	1.97	2.21	3.51	1.83	2.17	3.46	**1.80**
C_4H_9	4.95	M062X/def2QZVP	2	*2HO*-2HO	2.87	3.40	3.36	2.20	1.10	2.33	2.72	1.63	2.86	2.69	1.59	**2.82**
CF_3	2.75				−0.21	0.09	0.15	−0.80	−2.37	−0.62	−0.50	−2.07	−0.32	−0.43	−2.00	**−0.26**
CH_3O	5.29				3.83	4.20	4.19	3.20	2.08	3.23	3.57	2.46	3.60	3.56	2.44	**3.58**
CH_3	5.12				2.73	3.49	3.34	2.03	0.82	2.10	2.80	1.58	2.86	2.64	1.43	**2.71**
CN	1.79				−1.34	−1.05	−1.08	−1.93	−3.47	−1.63	−1.64	−3.18	−1.34	−1.68	−3.21	**−1.37**
$COCH_3$	2.23				−0.91	−0.56	−0.59	−1.41	−2.61	−1.19	−1.07	−2.27	−0.84	−1.10	−2.30	**−0.87**
I	3.78				1.95	2.19	2.14	1.51	0.35	1.59	1.74	0.58	1.82	1.70	0.54	**1.78**
NH_2	6.07				4.82	5.28	5.24	4.08	2.80	4.17	4.54	3.26	4.63	4.49	3.21	**4.58**
H	4.60				2.95	2.96	3.05	2.16	0.80	2.40	2.18	0.82	2.42	2.27	0.91	**2.51**
SO_3^-	3.17				1.66	1.71	1.74	0.68	−0.75	1.83	0.72	−0.70	1.88	0.75	−0.67	**1.91**
RMSE					2.25	1.93	1.94	2.87	4.16	2.65	2.55	3.84	2.31	2.56	3.85	**2.33**
C_4H_9	4.95	M062X/def2QZVP	3	2HONH-3HO	2.45	3.61	3.28	1.47	0.17	1.75	2.63	1.34	2.91	2.29	1.00	**2.58**
CF_3	2.75				0.58	1.48	1.15	−0.36	−2.22	−0.03	0.54	−1.32	0.86	0.21	−1.65	**0.54**
CH_3O	5.29				2.59	3.85	3.55	1.69	0.48	1.75	2.94	1.73	3.00	2.65	1.44	**2.71**
CH_3	5.12				2.85	3.56	3.40	1.94	0.69	2.04	2.64	1.40	2.74	2.49	1.24	**2.58**
CN	1.79				−0.61	0.17	0.01	−1.67	−3.45	−1.51	−0.88	−2.67	−0.73	−1.04	−2.83	**−0.89**
$COCH_3$	2.23				0.22	1.07	0.75	−0.69	−2.28	−0.31	0.16	−1.44	0.53	−0.16	−1.76	**0.21**
I	3.78				2.44	2.41	2.62	1.67	0.35	1.82	1.65	0.33	1.80	1.85	0.53	**2.01**
NH_2	6.07				5.03	5.14	5.17	4.01	2.72	4.21	4.13	2.84	4.33	4.15	2.86	**4.35**
H	4.60				1.88	2.74	2.54	1.01	−0.28	1.11	1.87	0.58	1.97	1.66	0.38	**1.77**
SO_3^-	3.17				1.46	2.25	2.04	0.33	−1.12	1.46	1.11	−0.33	2.25	0.91	−0.54	**2.04**
RMSE					2.15	1.37	1.56	3.08	4.51	2.82	2.31	3.75	2.06	2.50	3.93	**2.24**

Table S7. pK_a values of R-$PhNH_3^+$ calculated by revDSD-PBEP86-D3(BJ)/ma-def2QZVPP with models included three H_2O combined with amino group.

R group	With one H_2O near R	Experimental pK_a	Correction method			ΔpK_a		
			C0	C1	C2	C0	C1	C2
C_4H_9	No	4.95	4.86	6.02	5.69	0.09	1.07	0.74
CF_3	No	2.75	3.10	3.99	3.67	0.35	1.24	0.92
CH_3O	No	5.29	4.96	6.21	5.92	0.33	0.92	0.63
CH_3	No	5.12	5.23	5.93	5.78	0.11	0.81	0.66
CN	No	1.79	1.99	2.78	2.62	0.20	0.99	0.83
$COCH_3$	No	2.23	2.71	3.56	3.24	0.48	1.33	1.01
I	No	3.78	4.91	4.89	5.10	1.13	1.11	1.32
NH_2	No	6.07	7.27	7.39	7.41	1.2	1.32	1.34
H	No	4.60	4.35	5.21	5.00	0.25	0.61	0.4
SO_3^-	No	3.17	4.08	4.86	4.65	0.91	1.69	1.48
Average ΔpK_a obtained from models without H_2O near R						0.51	1.11	0.93
CF_3	Yes	2.75	2.98	3.01	2.71	0.23	0.26	0.04
CH_3O	Yes	5.29	5.39	6.02	5.91	0.10	0.73	0.62
$COCH_3$	Yes	2.23	2.70	3.26	2.98	0.47	1.03	0.75
*I	Yes	3.78	3.66	4.05	4.11	0.12	0.27	0.33
*NH_2	Yes	6.07	6.72	7.34	7.21	0.65	1.27	1.14
SO_3^-	Yes	3.17	4.52	4.84	4.71	1.35	1.67	1.54
*Average ΔpK_a obtained from models with H_2O near R (Bold and italic type)						0.36	0.87	0.71

*: With two symmetric H_2O molecules near the substitute of -I and -NH_2.

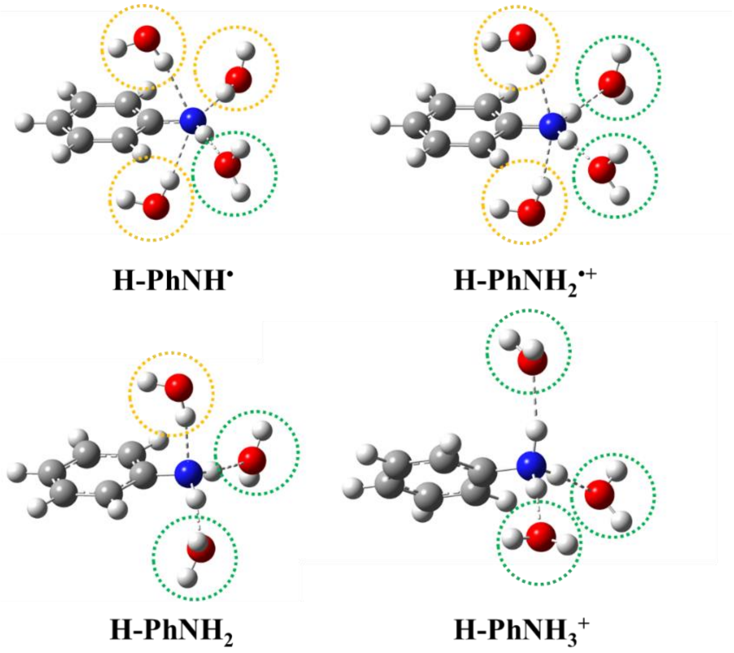

Figure S1. The potential positions of H_2O molecules in the models. H-PhNH$^•$, H-PhNH$_2^{•+}$, H-PhNH$_2$ and H-PhNH$_3^+$ are presented as examples. The H_2O molecules in green and orange circles are assumed to act as the electron (nitrogen) donor (labeled as NH) and hydrogen donor (labeled as HO), respectively.

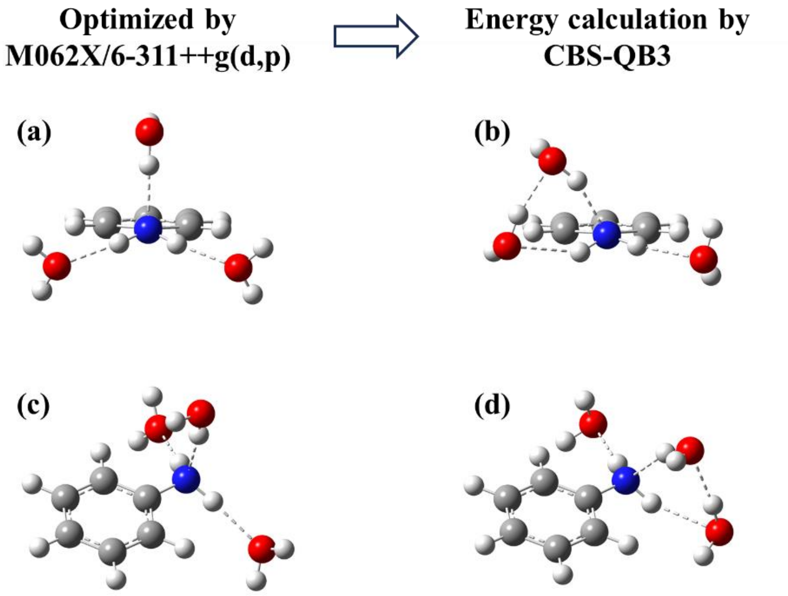

Figure S2. Comparison of H-PhNH$_2$ models with three H_2O molecules: (a,c) optimization based on the M062X(D3)/6-311++g(d,p) method, and (b,d) free energy calculation based on the CBS-QB3 method. (a,b) and (c,d) present two perspectives of the models, respectively.

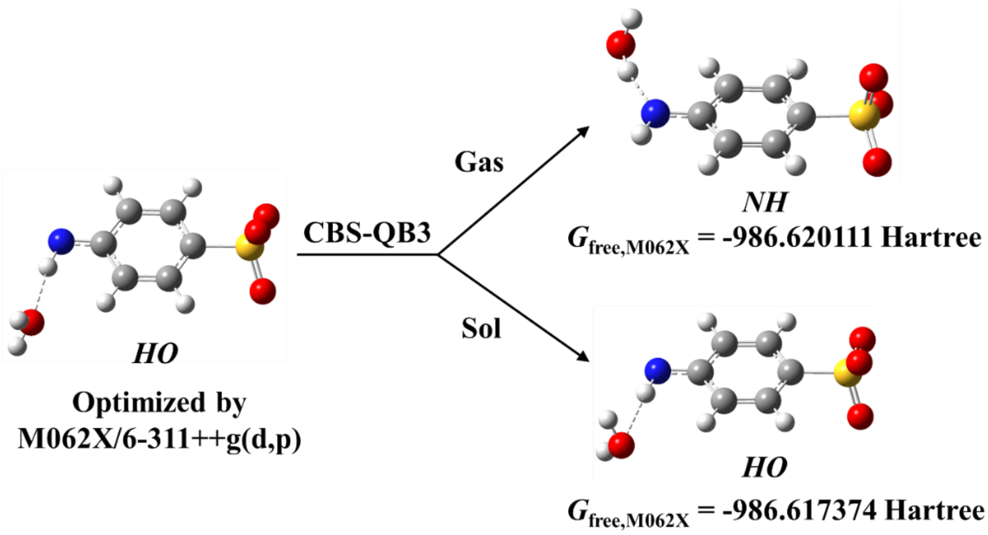

Figure S3. The changes in molecular structures after the calculation of *G*(gas) and *G*(sol) based on the CBS-QB3 method.

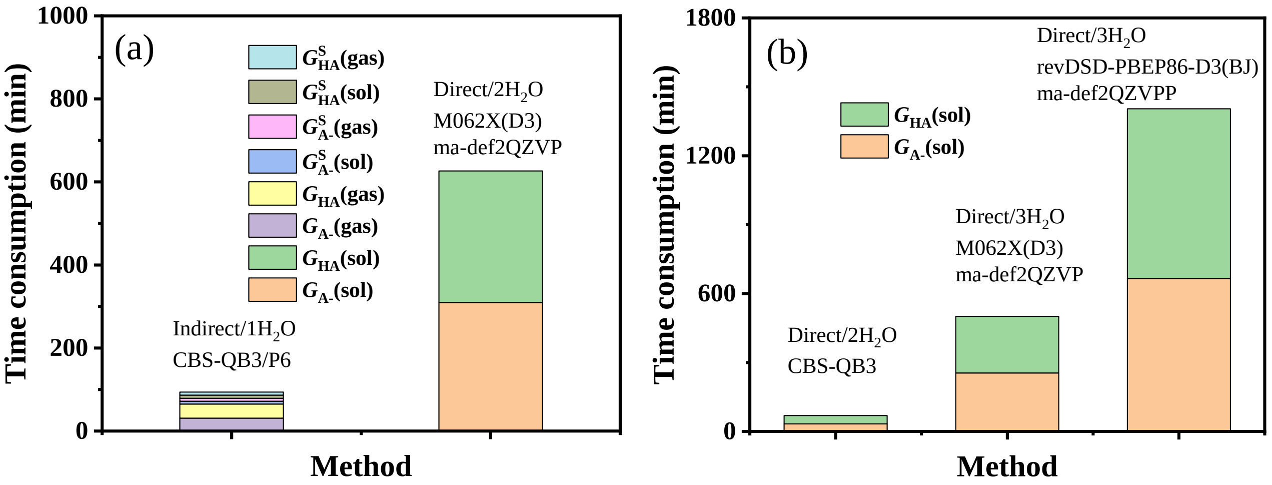

Figure S4. The calculation time for (a) H-$PhNH_2^{\bullet+}$ (indirect approach/CBS-QB3/C1/P6/1H_2O and direct approach/M062X(D3)/ma-def2QZVP/C1/2H_2O), and (b) H-$PhNH_3^{+}$ (direct approach/CBS-QB3/C1/2H_2O, direct approach/M062X(D3)/ma-def2QZVP/C1/3H_2O, and direct approach/M062X(D3)/revDSD-PBEP86-D3(BJ)/ma-def2QZVPP/3H_2O). The number of processors is 20.

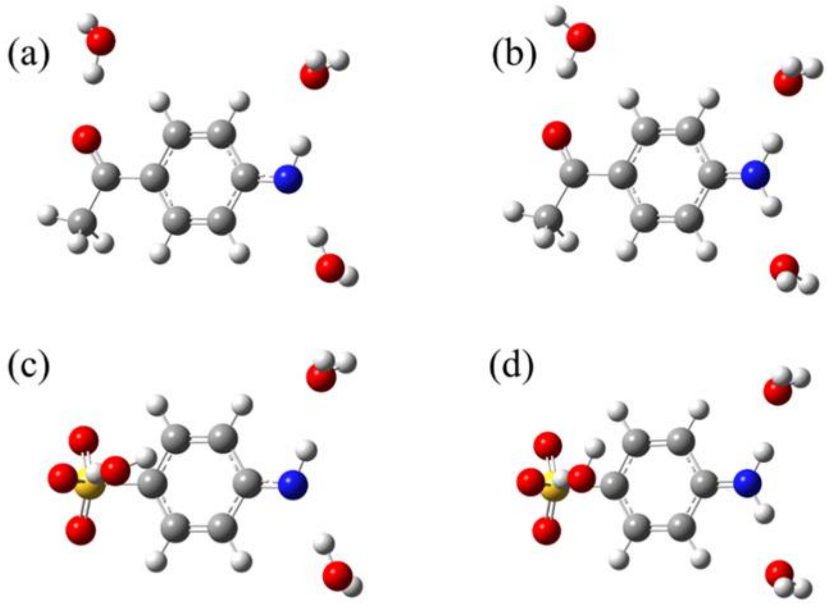

Figure S5. The optimized structures of (a) $COCH_3$-$PhNH^{\bullet}$, (b) $COCH_3$-$PhNH_2^{\bullet+}$, (c) SO_3^--$PhNH^{\bullet}$ and (d) SO_3^--$PhNH_2^{\bullet+}$ with an additional H_2O near =O.

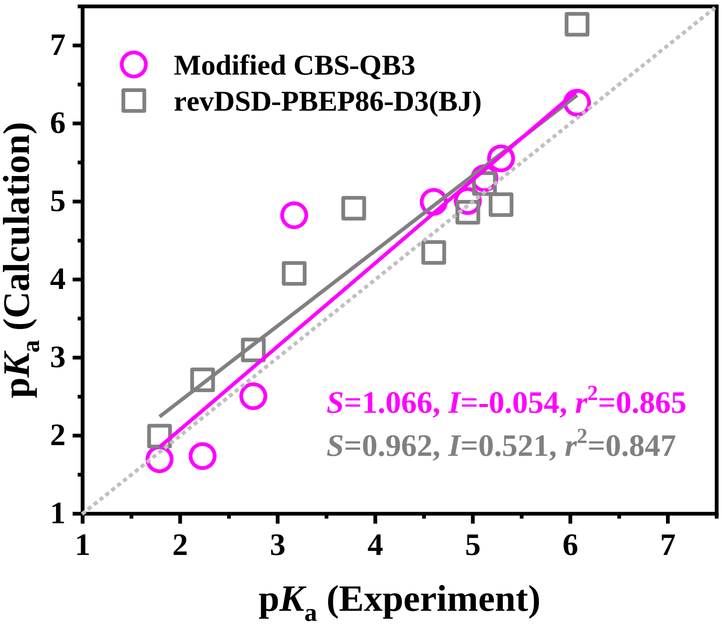

Figure S6. The performance of direct approach coupled with modified CBS-QB3 (model: $PhNH_2$ and $PhNH_3^+$ in *2HO* and 2HO, respectively) and revDSD-PBEP86-D3(BJ)/ma-def2QZVPP (model: $PhNH_2$ and $PhNH_3^+$ in *2HONH* and 3HO, respectively) methods on pK_a calculations of R-$PhNH_3^+$. The dotted line indicates the ideal results with S = 1 and I = 0.
